# Platelet Rubicon Bidirectional Regulation of GPVI and Integrin αIIbβ3 Signaling Mitigates Stroke Infarction Without Compromising Hemostasis

**DOI:** 10.1002/advs.202507509

**Published:** 2026-01-21

**Authors:** Xiaoyan Chen, Jingke Li, Yangyang Liu, Li Li, Xin Deng, Yilin Sheng, Xianyu Zhu, Xiao Jiang, Wei Li, Xueli Cai, Qiming Sun, Hu Hu

**Affiliations:** ^1^ Department of Pathology and Pathophysiology and Bone Marrow Transplantation Center of the First Affiliated Hospital Zhejiang University School of Medicine Hangzhou China; ^2^ Department of Biochemistry and Molecular Biology Zhejiang University School of Medicine Hangzhou China; ^3^ Institute of Hematology Zhejiang Engineering Laboratory for Stem Cell and Immunotherapy Zhejiang University Hangzhou China; ^4^ Lishui Central Hospital The Fifth Affiliated Hospital of Wenzhou Medical University Lishui Hospital of Zhejiang University Lishui China; ^5^ Department of Cardiology The First Affiliated Hospital of Zhengzhou University Zhengzhou China; ^6^ State Key Laboratory of Transvascular Implantation Devices Department of Cardiology of The Second Affiliated Hospital School of Medicine Zhejiang University Hangzhou China; ^7^ Heart Regeneration and Repair Key Laboratory of Zhejiang Province Hangzhou China

**Keywords:** cerebral ischemia‐reperfusion injury, glycoprotein VI, integrin αIIbβ3, Rubicon (Run domain protein as Beclin‐1 interacting and cysteine‐rich containing)

## Abstract

Inhibiting the platelet glycoprotein VI (GPVI) receptor is a promising strategy for reducing cerebral ischemia‐reperfusion injury (CIRI) without severe compromise of hemostasis, while targeting glycoprotein IIb/IIIa (integrin αIIbβ3) causes bleeding. The underlying cellular mechanism remains unclear. This study shows that megakaryocyte‐platelet‐specific deficiency of the autophagic protein Rubicon (Run domain protein as Beclin‐1 interacting and cysteine‐rich containing) accelerates stroke development and exacerbates cerebral hemorrhage. Rubicon interacts with Bruton's tyrosine kinase (Btk) to inhibit GPVI‐mediated thrombus formation, while it prevents αIIbβ3‐mediated selective autophagy and degradation of Btk to stabilize platelet thrombi. The expression of Rubicon in platelets is decreased in patients with acute ischemic‐reperfusion injury. A cell‐permeable peptide mimicking the Rubicon‐Btk interaction significantly reduces cerebral infarction volume in a mouse model. As Rubicon is dispensable for hemostasis but crucial in the reperfusion stage of CIRI, peptides mimicking its effects may offer a selective and safe therapeutic strategy.

## Graphical abstract

This study identifies Rubicon as a key platelet protein that bidirectionally regulates GPVI and integrin αIIbβ3 signaling. Platelet Rubicon protects against cerebral ischemia‐reperfusion injury by limiting infarction without increasing hemorrhage. A cell‐penetrating peptide mimicking Rubicon function reduces ischemic injury, presenting a novel therapeutic strategy for stroke.

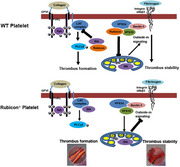

## 1 Introduction

Platelets are the cornerstone of hemostasis and also the major culprits in pathological thrombosis. Reflecting a “double‐edged sword” effect, existing antiplatelet therapies consistently suffer from bleeding risk [1]. A typical disease representing this treatment dilemma is cerebral ischemia‐reperfusion injury (CIRI); patients may deteriorate into intracerebral hemorrhage (ICH) after antiplatelet therapy [2]. Therefore, it is critical to rigorously investigate platelet activation mechanisms to distinguish their physiological and pathological roles. Recent studies have revealed that pathological thrombosis, such as CIRI, can be distinguished from hemostasis via platelet‐specific receptors. Blocking glycoprotein VI (GPVI), the major platelet receptor for collagen, protects animals from CIRI in a model of middle cerebral artery occlusion (tMCAO) [3]. In contrast, blockade of glycoprotein IIb/IIIa (integrin αIIbβ3), the key adhesion molecule responsible for platelet homotypic aggregation, has no effect on stroke size but rather increases the incidence of ICH and mortality in the same tMCAO model [3]. GPVI is responsible for the rapid initiation of thrombus formation, whereas αIIbβ3 serves to form the bridge between platelets and maintain the stability of the thrombi. Despite the fact that GPVI and αIIbβ3 are both crucially involved in thrombosis, the paradoxical outcomes of CIRI and ICH caused by the differential targeting of these two receptors remain poorly explained. Facing the rapid increase in the prevalence of stroke worldwide and the limited treatment options [4], an in‐depth understanding of the intracellular events that underscore the effects of GPVI and αIIbβ3 in CIRI and ICH is urgently needed.

Autophagy is an evolutionarily conserved catabolic pathway that sequesters cytoplasmic proteins and organelles for lysosomal degradation [5]. Autophagy in platelets has been suggested as an important player in ischemia‐reperfusion injury [6, 7, 8]. Evidence garnered from mice deficient in key autophagic regulators, such as Beclin‐1 [9], ATG7 [10], ATG5 [11], and VPS34 (also known as PK3C3) [12, 13], has substantiated the functional importance of autophagic proteins in platelet activation [6, 9, 10, 11, 12, 13, 14, 15, 16]. Noticeably, as illustrated in the previous studies [12], VPS34 promotes GPVI‐mediated platelet activation, yet is either not involved in the pathway mediated by αIIbβ3 (outside‐in signaling) [12] or negatively regulates it [13]. Together, these observations indicate that autophagic proteins, especially those related to VPS34, may harbor the key mechanism to differentially regulate GPVI and αIIbβ3 signaling in the context of ischemia‐reperfusion injury. However, it is currently unclear how autophagy, as a conserved catabolic process, is integrated into the platelet activation process. Equally unknown is whether autophagic proteins may be specifically targeted by thrombotic or bleeding disorders.

Run domain protein as Beclin‐1 interacting and cysteine‐rich containing (Rubicon) is a cytoplasmic protein [17]. Well‐known for its role in the negative regulation of VPS34 complex and autophagy [18], Rubicon has been revealed as an important regulator of ischemia‐reperfusion injury in multiple tissues [19, 20, 21, 22]. For example, cardiomyocyte‐expressed Rubicon was found to exacerbate ischemia‐reperfusion injury in a mouse model of myocardial infarction [19, 21]. Endothelial cell‐expressed Rubicon reduces the viability of endothelial cells and leads to the breakdown of the blood–brain barrier (BBB) in a model of tMCAO [20]. Moreover, although autophagy regulation is the most extensively reported mechanism of Rubicon, Rubicon also harbors autophagy‐independent mechanisms, such as targeting of CBM complex (CARD9/BCL10/MALT1) in cytokine production [23], interacting with Rab7 in endosomal maturation [24, 25], and regulating NOX2 in phagocytosis [26]. Given the imperative to understand how platelet receptors are differentially regulated in CIRI, Rubicon emerges as an important candidate for three reasons: (1) as the primary VPS34 inhibitor, Rubicon is uniquely positioned to elucidate the mechanisms underlying the differential regulation of GPVI versus αIIbβ3 signaling by VPS34. This distinction is crucial, considering therapeutic potential of GPVI as a stroke‐specific target without bleeding risks [18]; (2) Rubicon's dual autophagy‐dependent and ‐independent mechanisms [23, 24, 25, 26, 27] may reveal whether this differential regulation occurs through canonical autophagy or alternative signaling pathways; and (3) established roles of Rubicon in ischemia‐reperfusion injury across vascular tissues [19, 20, 21, 22] suggest it may similarly modulate platelet‐mediated thrombosis. Nevertheless, whether Rubicon is expressed in platelets, how it differentially regulates GPVI versus αIIbβ3 signaling, and whether it contributes to cerebral ischemia‐reperfusion injury remain unknown.

In this study, with the generated megakaryocyte/platelet‐specific Rubicon‐deficient mice, we found that Rubicon plays important roles in platelet‐mediated CIRI and ICH through mechanisms encompassing GPVI signaling, integrin αIIbβ3 outside‐in signaling, and autophagy. The significance and therapeutic potential of our findings were demonstrated by the successful reduction of CIRI by a Rubicon‐derived cell‐penetrating peptide designed to modulate the interaction of Rubicon and its partners.

## 2 Results

### 2.1 Megakaryocyte/Platelet‐Specific Rubicon Deficiency Results in a Paradoxical Hemostatic Phenotype

**FIGURE 1:**
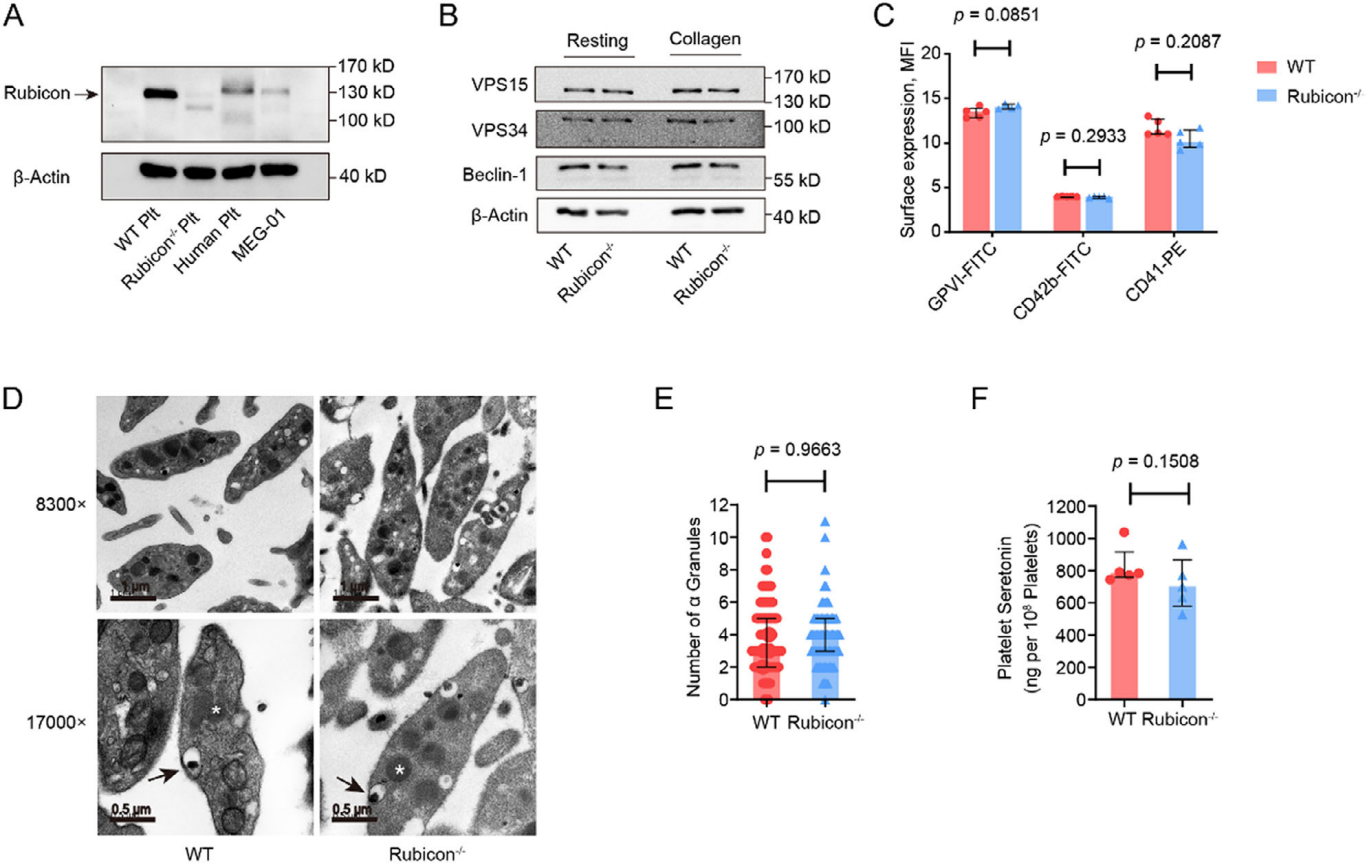
Platelet Rubicon deficiency does not alter platelet morphology or granule content. (A) Immunoblot analysis of Rubicon expression in platelets from human, WT and *Rubcn^f/f^ PF4‐Cre^+^* (Rubicon^−/−^) mice, MEG ‐ 01 cell line. (B) Expression of VPS15, VPS34, and Beclin‐1 in platelets from WT and *Rubcn^f/f^ PF4‐Cre^+^* mice. (C) Flow cytometric analysis of platelet surface GPVI, CD42b, and CD41 expression. Data shown as mean fluorescence intensity (MFI, Student *t* test). (D) Representative electron microscopic images of platelet ultrastructure. Scale bars: upper, 1 µm; lower, 0.5 µm. Arrowhead represents dense granule; asterisk represents α ‐ granule. (E) Quantification of α‐granules per platelet (WT: *n* = 81, Rubicon^−/−^: *n* = 79, two‐tailed Mann–Whitney *U* test). (F) Serotonin content in platelets from WT and *Rubcn^f/f^ PF4‐Cre^+^* mice (two‐tailed Mann–Whitney *U* test). These data are shown as median with interquartile range.

Rubicon is expressed in both human and mouse platelets, as well as in the megakaryocytic cell line MEG‐01 (Figure 1A). To define the role of Rubicon in platelet activation, hemostasis, and thrombosis, we constructed megakaryocyte/platelet‐specific Rubicon‐deficient (*Rubcn^f/f^ PF4‐Cre^+^*) mice (Figure 1A). These mice exhibited normal viability and fertility with no spontaneous bleeding or thrombotic events. Basic hematological parameters, including platelet count, red blood cell count, and white blood cell count, were comparable between knockout and wild‐type mice, with only a slight increase in mean platelet volume (Table 1).

**TABLE 1:** Hematologic analysis. Data are shown as mean ± SEM. No abnormalities or significant differences between WT and *Rubcn^f/f^ PF4‐Cre^+^* mice were found for hematologic parameters (*n* = 10; two‐tailed Mann–Whitney *U* test). MPV, mean platelet volume; RBC, red blood cell; WBC, white blood cell.

	WT	Rubicon^−/−^	*P*
**RBCs, × 10^12^/L**	8.550 ± 0.2486	8.507 ± 0.2204	1.0000
**WBCs, × 10^9^/L**	2.790 ± 0.2248	2.423 ± 0.2807	0.3256
**Platelets, × 10^9^/L**	714.9 ± 26.83	683.4 ± 21.91	0.5288
**Hematocrit, ％**	40.38 ± 1.023	40.64 ± 1.139	0.7912
**Hemoglobin, g/dL**	12.32 ± 0.2412	12.44 ± 0.3721	0.5444
**MPV, fL**	5.360 ± 0.03712	5.530 ± 0.04955	0.0162*

Rubicon deficiency did not alter the expression of class III PI3K complex components (Figure 1B) or platelet surface glycoproteins (Figure 1C). Ultrastructural analysis revealed normal platelet morphology, including the open canalicular system and α‐granule distribution (Figure 1D,E). Serotonin content was also comparable between wild‐type and Rubicon‐deficient platelets (Figure 1F). In addition, Rubicon^−/−^ megakaryocytes (MKs) exhibited normal ploidy levels, normal sizes, and nuclear sizes (Figure S1). These results indicated that Rubicon is dispensable for normal platelet development and granule formation.

**FIGURE 2:**
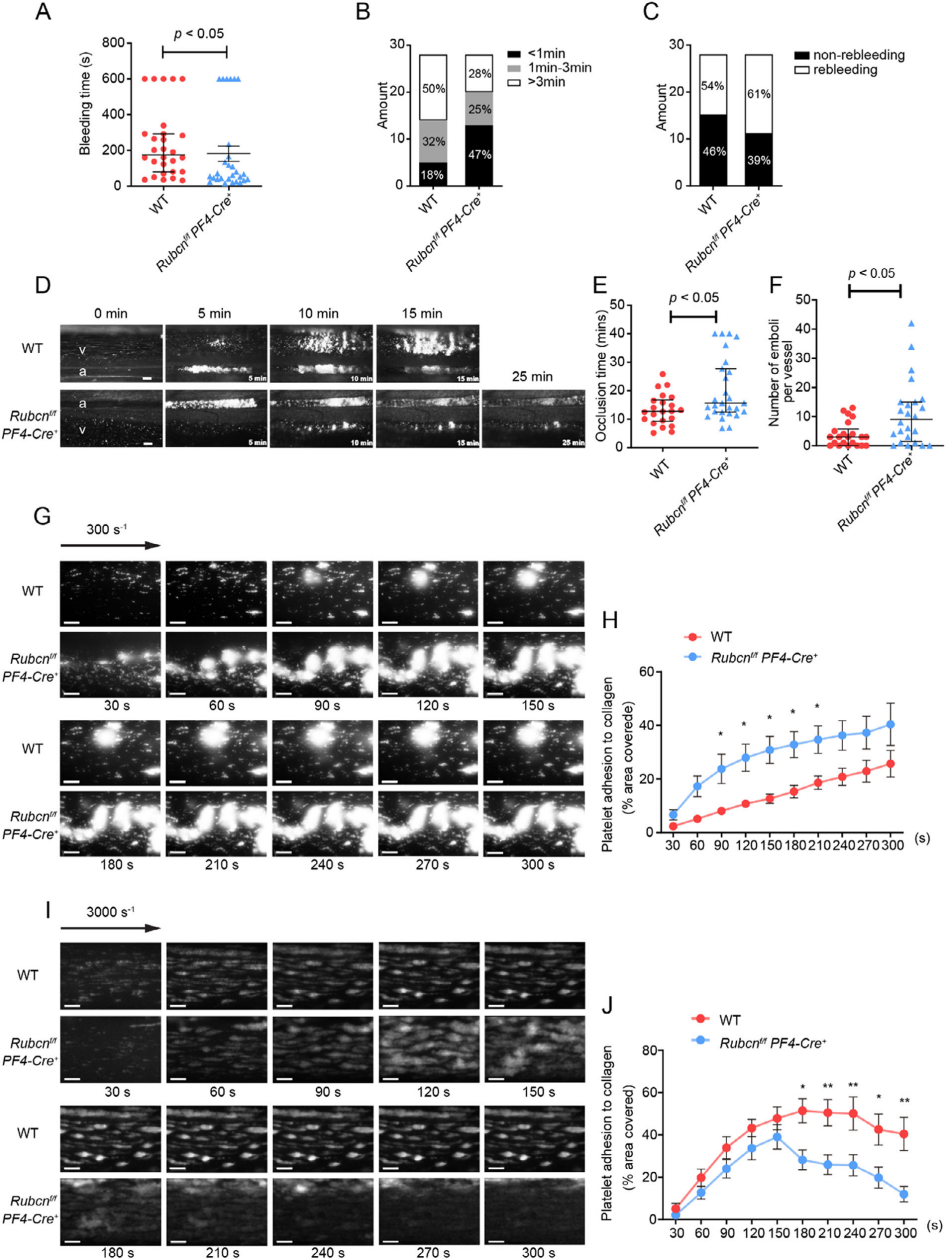
Rubicon deficiency mildly reduces bleeding time but impairs thrombus stability in vivo and under flow. (A) Tail bleeding time in WT (●) and *Rubcn^f/f^ PF4‐Cre^+^* (▲) mice. (B) Distribution of bleeding times categorized as >3 min, 1–3 min, or <1 min. (C) Quantification of re‐bleeding times. (D) Representative time‐lapse images of FeCl_3_‐induced thrombus formation in mesenteric arterioles. a, arteriole; v, venule. Scale bar, 100 µm. (E) Dot plot showing occlusion times for arterioles as a result of FeCl_3_‐induced thrombosis in WT (●) and *Rubcn^f/f^ PF4‐Cre^+^* mice (▲). The results are shown as median with interquartile range (two‐tailed Mann–Whitney *U* test). (F) Dot plot showing the number of emboli (> 20 µm) shed from the artery injury site during thrombosis formation in WT mice (●) and *Rubcn^f/f^ PF4‐Cre^+^* mice (▲). The results are shown as median with interquartile range (two‐tailed Student *t*‐test). (G) Mepacrine‐labeled whole blood from WT (*n* = 8) and *Rubcn^f/f^ PF4‐Cre^+^* (*n* = 6) mice was perfused through a fibrillar collagen‐coated surface. Representative images of thrombus formation under low shear (300 s^−^^1^) in collagen‐coated microfluidic chambers. Scale bar, 50 µm. (H) Quantification of area coverage over time at 300 s^−^^1^. Statistical significance was evaluated with a two‐way ANOVA test (*p < 0.05). The results are shown as mean ± SEM. (I) Mepacrine‐labeled whole blood from WT (*n* = 8) and *Rubcn^f/f^ PF4‐Cre^+^* (*n* = 9) mice was perfused through a fibrillar collagen‐coated surface at a shear rate of 3000 s^−1^. Representative images and time courses of thrombus formation. Scale bar, 50 µm. (J) Quantification of area coverage over time at 3000 s^−^^1^. Statistical significance was evaluated with a two‐way ANOVA test (*p < 0.05, **p < 0.01). The results are shown as mean ± SEM.

Despite normal platelet development, Rubicon deficiency resulted in contrasting effects on hemostasis and thrombosis. In tail bleeding assays, *Rubcn^f/f^ PF4‐Cre^+^* mice exhibited significantly shortened bleeding times (182.0 ± 88.17 s vs. 237.4 ± 74.75 s; Figure 2A), with nearly half (47%) showing bleeding times under 1 min compared to only 17% in wild‐type controls (Figure 2B). In addition, *Rubcn^f/f^ PF4‐Cre^+^* mice displayed a slightly higher incidence of rebleeding (61% vs. 54%, Figure 2C). Paradoxically, in FeCl_3_‐induced mesenteric arteriole injury models, Rubicon‐deficient mice displayed prolonged time to stable occlusion (Figure 2D,E) and a nearly threefold increase in detaching emboli (10.86 ± 3.21 vs. 3.31 ± 1.12 per vessel injury; Figure 2F, Videos S1 and S2), suggesting impaired thrombus stability.

This paradox was further explored using whole blood perfusion assays under varying shear conditions. At low shear (300 s^−^^1^), Rubicon‐deficient platelets formed larger thrombi on collagen surface (Figure 2G,H; Videos S3 and S4), whereas at high shear (3000 s^−^^1^), they initially formed normal thrombi that subsequently became highly unstable (Figure 2I,J; Video S5). These findings indicate that Rubicon limits initial thrombus formation under low shear conditions but is essential for maintaining thrombus stability under high shear, suggesting differential regulation of distinct platelet activation pathways.

### 2.2 Rubicon Differentially Regulates GPVI and Integrin αIIbβ3 Signaling Pathways

**FIGURE 3:**
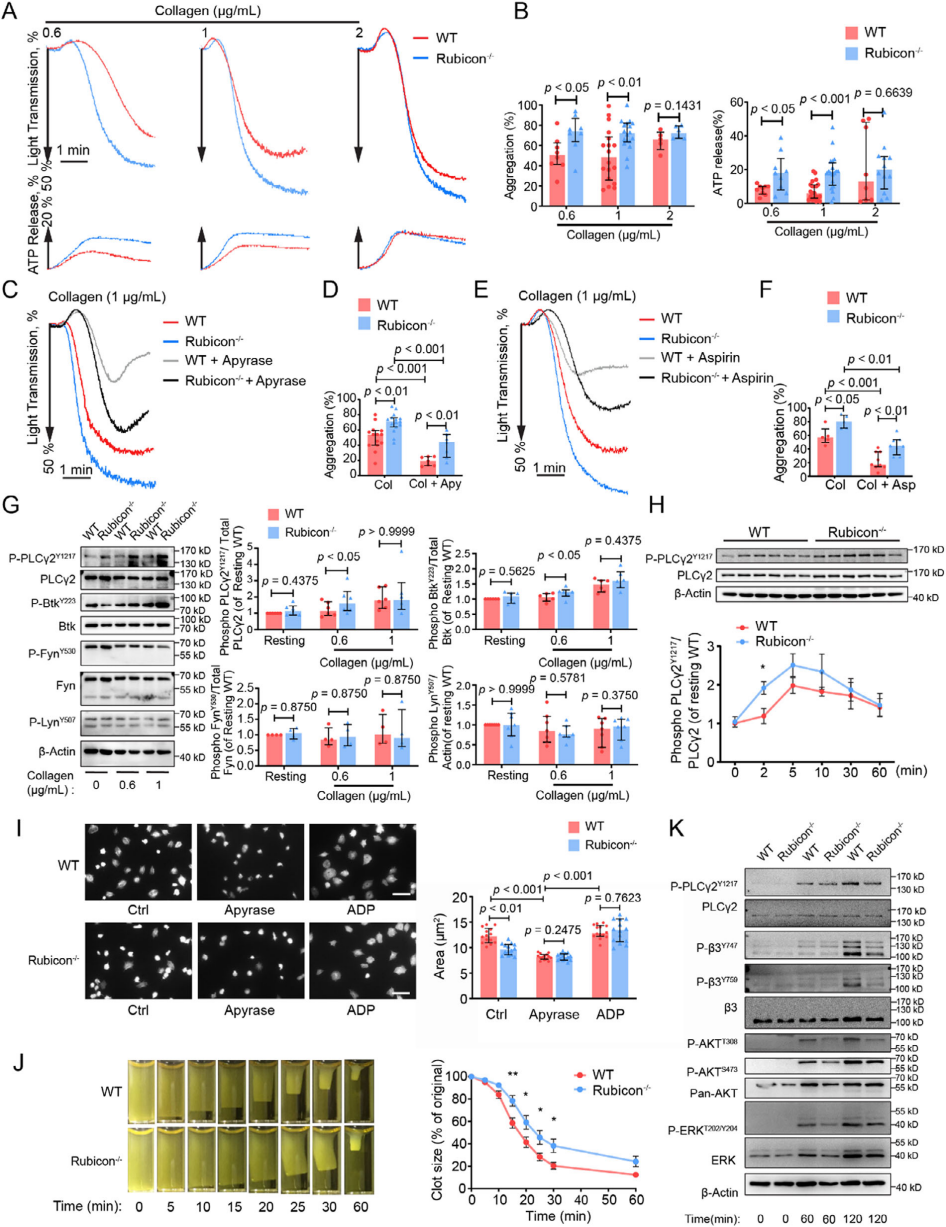
Rubicon‐deficient platelets exhibit enhanced GPVI‐mediated aggregation and secretion but impaired αIIbβ3 outside‐in signaling. (A) Aggregation and ATP secretion of washed platelets stimulated with indicated concentrations of collagen. Aggregation and ATP secretion (with luciferase) were assessed with a Chrono‐log lumi‐aggregometer under stirring at 1200 rpm. (B) Quantification of aggregation (left) and ATP release (right) depicted in panel A. The results are shown as median with interquartile range (two‐tailed Mann–Whitney *U* test). (C) Aggregation after apyrase (0.1U/mL) pre‐treatment, followed by collagen stimulation. (D) Quantification of aggregation depicted in panel C. The results are shown as median with interquartile range (two‐tailed Mann–Whitney *U* test). (E) Aggregation after aspirin pre‐treatment, followed by collagen stimulation. (F) Quantification of aggregation depicted in panel E. The results are shown as median with interquartile range (two‐tailed Mann–Whitney *U* test). (G) Western blot analysis of GPVI pathway signaling proteins after collagen stimulation. Quantification of phosphorylated PLCγ2^Y1217^, Btk^Y223^, Fyn ^Y530^, Lyn ^Y507^ in WT and Rubicon^−/−^ platelets stimulated with collagen. The results are shown as median with interquartile range (Wilcoxon signed‐rank test). (H) Time‐course analysis of PLCγ2^Tyr1217^ phosphorylation in WT and Rubicon^−/−^ platelets stimulated with collagen. The results are shown as mean ± SEM (two‐tailed Mann–Whitney *U* test). (I) Spreading of WT and Rubicon^−/−^ platelets on immobilized fibrinogen in the presence or absence of apyrase (0.1U/mL) or ADP (1 µm), scale bar: 5 µm. Quantification of the areas of spread WT and Rubicon^−/−^ platelets. The results are shown as median with interquartile range (two‐tailed Mann–Whitney *U* test). (J) Platelets from WT (*n* = 6) and *Rubcn^f/f^ PF4‐Cre^+^* (*n* = 7) mice were resuspended with human platelet‐poor plasma at a concentration of 4 × 10^8^ mL^−1^, and recombined plasma was stimulated to coagulate with thrombin (0.4 U/mL), then photographed at different time points. Statistical significance was evaluated with a two‐way ANOVA test, and the results are shown as mean ± SEM (*p < 0.05, **p < 0.01). (K) Western blot analysis of outside‐in signaling (phosphorylated β3, AKT, PLCγ2, ERK) during platelet spreading on fibrinogen.

To elucidate the molecular basis for the observed hemostatic phenotype, we examined platelet aggregation and signaling responses to various agonists. Rubicon‐deficient platelets showed significantly enhanced aggregation and dense granule secretion in response to low concentrations of collagen (0.6, 1 µg mL^−1^) but not to higher concentrations (2 µg mL^−1^) (Figure 3A,B). In contrast, responses to thrombin, ADP, or U46619 were unaltered (Figure S2), indicating pathway‐specific effects. Moreover, the levels of TXB_2_ and VASP phosphorylation induced by collagen stimulation in Rubicon^−/−^ platelets were similar to those observed in WT platelets (Figure S3). Pre‐treatment with apyrase or aspirin did not abolish the enhanced collagen responses (Figure 3C–F), suggesting a direct effect on the GPVI pathway rather than secondary amplification through dense granule secretion or thromboxane A_2_.

Biochemical analyses revealed enhanced phosphorylation of Bruton's tyrosine kinase (Btk) and PLCγ2 in Rubicon‐deficient platelets following collagen stimulation, while phosphorylation of early signaling molecules (Lyn, Fyn, Syk, LAT, SLP‐76) remained unchanged (Figure 3G; Figure S4). Temporal analysis demonstrated sustained enhancement of PLCγ2 phosphorylation throughout the aggregation response (Figure 3H), confirming hyperactivation of the GPVI pathway. In stark contrast to its inhibitory effect on GPVI signaling, Rubicon positively regulated integrin αIIbβ3 outside‐in signaling. Rubicon‐deficient platelets showed significantly reduced spreading area on immobilized fibrinogen (9.687 ± 0.2929 µm^2^ vs. 12.34 ± 0.3921 µm^2^), which was normalized by adding exogenous ADP (Figure 3I). Clot retraction, which is also dependent on αIIbβ3 outside‐in signaling, was significantly delayed in Rubicon‐deficient platelets (Figure 3J). Biochemical analysis revealed reduced phosphorylation of β3 (Tyr^7^ ^4^ ^7^, Tyr^7^ ^5^ ^9^), AKT (Thr^3^ ^0^ ^8^, Ser^4^ ^7^ ^3^), PLCγ2 (Tyr^1^ ^2^ ^1^ ^7^), and ERK (Thr^2^ ^0^ ^2^/Tyr^2^ ^0^ ^4^) during fibrinogen spreading (Figure 3K), confirming impaired αIIbβ3 outside‐in signaling. These findings establish Rubicon as a bidirectional regulator that inhibits GPVI‐mediated activation while promoting αIIbβ3‐mediated outside‐in signaling, explaining the paradoxical hemostatic phenotype observed in vivo.

### 2.3 Molecular Mechanisms for Dual Regulatory Functions of Rubicon

**FIGURE 4:**
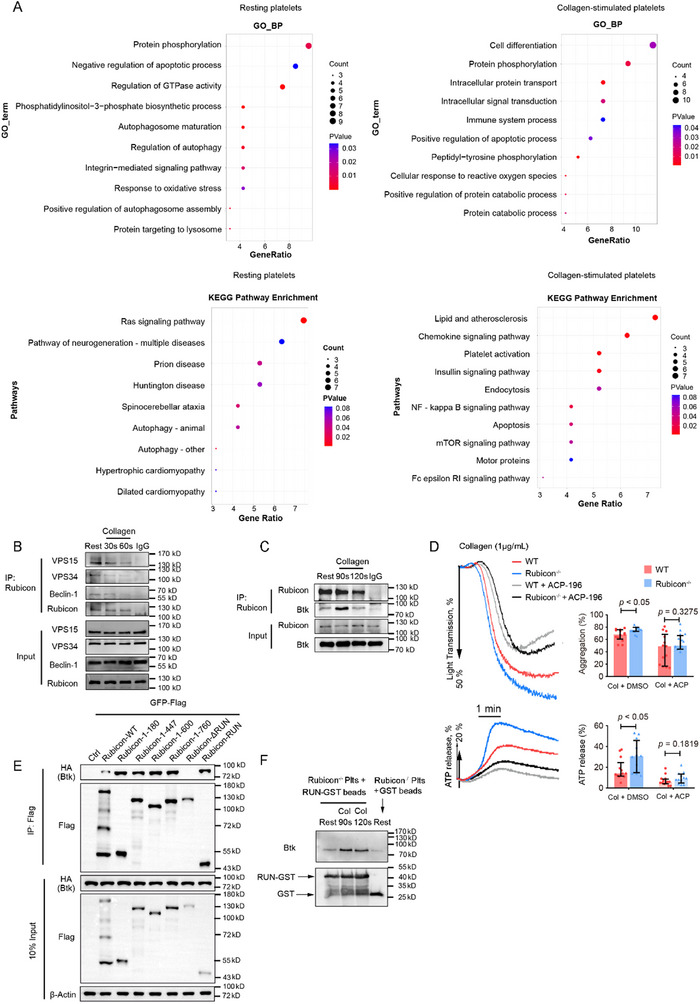
Rubicon dissociates from the VPS34 complex and interacts with Btk upon GPVI activation. (A) GO pathway and KEGG pathway were enriched by the database for annotation, visualization, and integrated discovery (DAVID) from tandem mass spectrometry data. (B) Co‐immunoprecipitation showing Rubicon association with VPS15, VPS34, and Beclin‐1 in resting platelets. (C) Co‐IP showing Rubicon‐Btk interaction after collagen stimulation. (D) Platelet aggregation and ATP secretion after Btk inhibition (ACP‐196) (0.5 µM) and collagen stimulation. Quantification of platelet aggregation (top) and ATP release (bottom). The results are shown as median with interquartile range (two‐tailed Mann–Whitney *U* test). (E) Mapping of Rubicon domains required for Btk binding using FLAG‐tagged Rubicon fragments co‐expressed with HA‐Btk in HEK293T cells. (F) GST pull‐down assay confirming RUN domain‐Btk interaction in resting and activated Rubicon^−/−^ platelet lysates.

To elucidate the molecular mechanisms underlying Rubicon's dual regulatory functions, we performed immunoprecipitation followed by mass spectrometry analysis of Rubicon‐interacting proteins in resting and collagen‐stimulated platelets (Table S1). Gene Ontology and KEGG pathway analyses revealed that in resting platelets, Rubicon associates primarily with autophagy‐related proteins, while in collagen‐stimulated platelets, it interacts with proteins involved in cell differentiation, intracellular protein transport, and signal transduction (Figure 4A).

In resting platelets, Rubicon co‐precipitated with VPS34, VPS15, and Beclin‐1 (Figure 4B; Table S1), consistent with its established role as a negative regulator of the class III PI3K complex. Upon collagen stimulation, Rubicon dissociated from these proteins and interacted instead with Btk (Figure 4C; Table S1), a key signaling molecule in the GPVI pathway. Rubicon deficiency increased PI(3)P production in both resting and stimulated platelets (Figure S5A), confirming its inhibitory effect on VPS34 activity [18, 28]. Consistent with the previous findings [12], Rubicon deficiency also enhanced phoxp40 phosphorylation (marker for NOX assembly) and ROS generation upon GPVI stimulation (Figure S5B,C), scavenging ROS with N‐acetyl‐cysteine (NAC) did not abolish the enhanced aggregation (Figure S5D), albeit NAC caused a similar decrease of platelet aggregation in both Rubicon^−/−^ and WT platelets, suggesting that an increased ROS production upon Rubicon deficiency was not the primary mechanism of enhanced GPVI signaling.

The crucial role of Btk in mediating Rubicon's inhibitory effect on GPVI signaling was demonstrated by the addition of the Btk inhibitor Acalabrutinib (ACP‐196), which eliminated the aggregation differences between wild‐type and Rubicon‐deficient platelets (Figure 4D). To identify the Btk‐binding domain of Rubicon, we generated various Rubicon polypeptide fragments, including: (1) deletions of the RUN domain (△RUN, aa 180‐972) and the FYVE‐like domain (△FYVE‐like, aa 1‐760); (2) truncated segments (aa 1‐180, 1‐447, 1‐600); and (3) the isolated RUN domain (aa 49‐180). Immunoprecipitation revealed that the RUN domain (aa 49–180) is necessary and sufficient for Btk interaction (Figure 4E). Further confirmation of the interaction between RUN domain and Btk was performed by a GST pull‐down assay by incubating GST‐RUN recombinant protein with Rubicon^−/−^ platelets lysate. Compared with resting platelets lysate, the RUN domain of Rubicon has a stronger interaction with Btk in activated platelet lysate (Figure 4F). Further mapping identified the N‐terminal region of Btk (aa 1‐167), containing the PH domain, as the binding interface with Rubicon (Figure S6).

**FIGURE 5:**
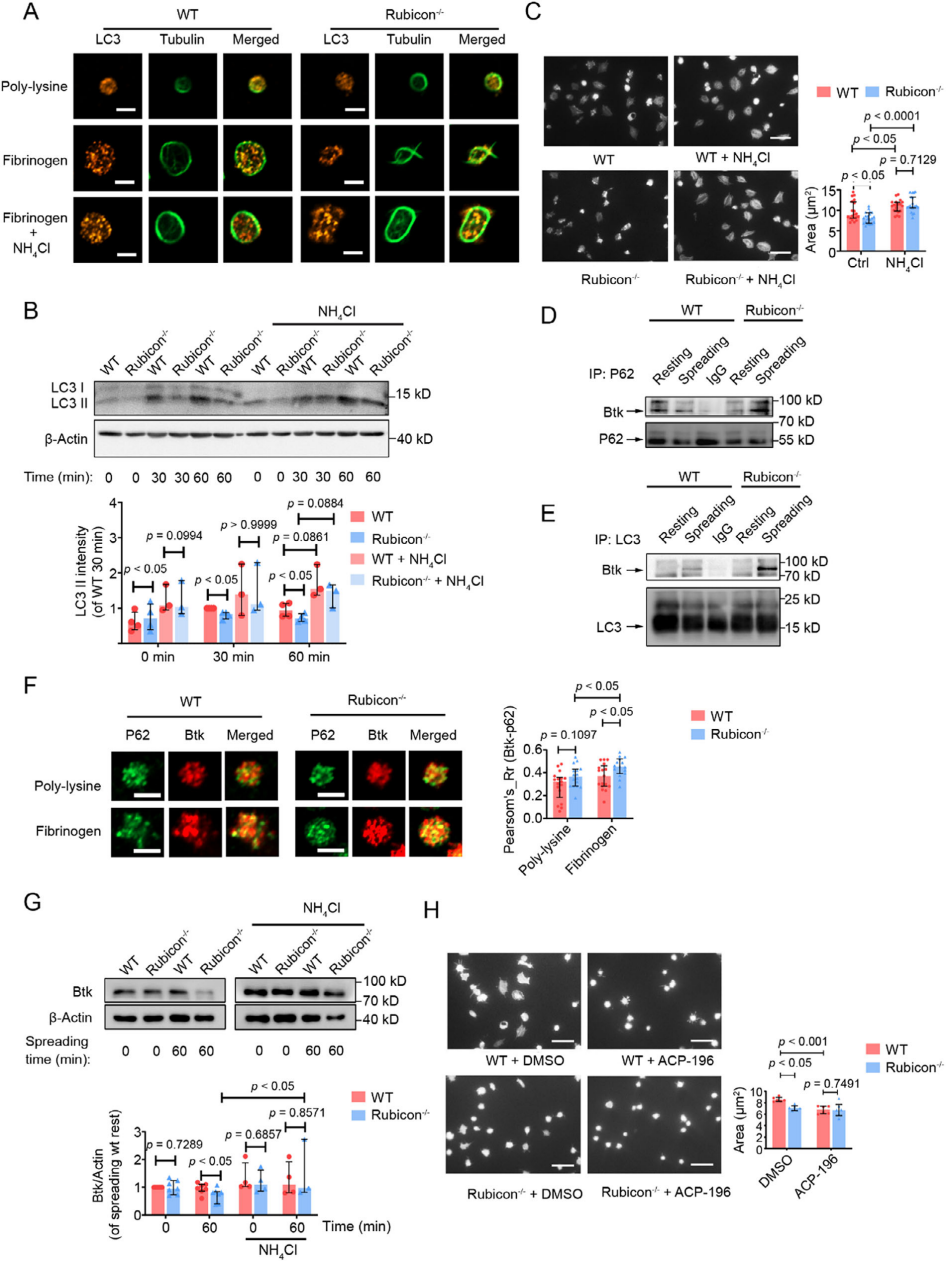
Rubicon promotes αIIbβ3 outside‐in signaling by preventing autophagic Btk degradation. (A) Confocal images of LC3 puncta in platelets spread on poly‐L‐lysine (20 µg/mL) or fibrinogen (20 µg/mL), with or without NH_4_Cl (20 mM). Scale bar, 2 µm. (B) WT and Rubcion^−/−^ platelets spread on fibrinogen were treated with NH_4_Cl (20 mM) and lysed at the indicated time points. Analysis and quantification of LC3‐II levels in platelets are shown as median with interquartile range (two‐tailed Mann–Whitney *U* test). (C) Platelet spreading on fibrinogen with or without NH_4_Cl (20 mM); representative images and quantification of spread area. Scale bar, 5 µm. (D) Co‐immunoprecipitation of Btk with p62 in resting and fibrinogen‐stimulated platelets. (E) Co‐immunoprecipitation of Btk with LC3 in resting and fibrinogen‐stimulated platelets. (F) Confocal microscopy for p62‐Btk colocalization in spread platelets. Scale bar, 2 µm. Quantification of p62‐Btk colocalization using Pearson's coefficient. (G) Btk protein levels in platelets after spreading on fibrinogen for 60 min in the presence or absence of NH_4_Cl (20 mM), analyzed by immunoblot and quantified. (H) Platelet spreading on fibrinogen with Btk inhibitor (ACP‐196) (0.5 µm) or vehicle; representative images and quantification. Statistical significance was evaluated with a two‐tailed Mann–Whitney *U* test; the data are shown as median with interquartile range.

To investigate the mechanism of the positive regulation of Rubicon in αIIbβ3 outside‐in signaling, we examined autophagy during platelet spreading on fibrinogen. Rubicon^−/−^ platelets formed fewer LC3‐positive puncta and showed reduced LC3‐II formation compared to WT platelets when spread on fibrinogen (Figure 5A,B). Treatment with NH_4_Cl, which blocks autophagic flux by neutralizing lysosomal pH [29], abolished these differences (Figure 5A,B) and normalized the spreading area of Rubicon‐deficient platelets (Figure 5C). In addition, bafilomycin A1, which blocks autophagic flux by inhibiting vacuolar‐type H^+^‐ATPase (V‐ATPase), restored LC3‐II formation in Rubicon^−/−^ platelets to levels similar to those in WT platelets (Figure S7A). These data indicate that Rubicon promotes outside‐in signaling by inhibiting autophagy.

To elucidate the functional stage of Rubicon during autophagy, we conducted LC3 II immunofluorescence and LysoSensor staining in platelets on fibrinogen. The results showed that Rubicon deficiency reduced the number of LC3 II^+^ puncta and increased the number of LAMP2^+^ LC3 II^+^ puncta (Figure S7B). As lysosomal acidification was evaluated using LysoSensor Green DND‐189 staining, comparable fluorescence intensity was observed between Rubicon^−/−^ and WT platelets, indicating that Rubicon deficiency does not affect lysosomal acidity (Figure S7C). Therefore, Rubicon deficiency may influence autophagic flux in platelets mainly through an enhanced autophagosome–lysosome fusion, as previously reported in other cell types [30]. The reduced number of LC3 II^+^ puncta by Rubicon deficiency may also be a result of this enhanced autophagosome–lysosome fusion and degradation, similar to the conclusion drawn in the previous study [30].

We then examined whether selective autophagic degradation of signaling molecules might explain the impaired outside‐in signaling. Because proteins degraded by selective autophagy are tagged with ubiquitin [31], we profiled by 4D Mass spectrometry the ubiquitination of proteins in platelets stimulated with or without Mn^2+^ to induce integrin outside‐in signaling. Profiling of protein ubiquitination in platelets stimulated with Mn^2^^+^ revealed that Btk was ubiquitinated at Lys^1^ ^2^ (Figure S8), with 1.5‐fold higher ubiquitination in Rubicon‐deficient platelets (Table 2). During spreading on fibrinogen, Rubicon‐deficient platelets showed increased Btk‐p62‐LC3II interaction (Figure 5D,E) and stronger Btk‐p62 co‐localization (Figure 5F). Importantly, Btk protein levels decreased significantly in Rubicon‐deficient platelets after 60 min of spreading (Figure 5G; Figure S9). This reduction was prevented by NH_4_Cl and Bafilomycin A1, but not by MG‐132, suggesting autophagic rather than proteasomal degradation is responsible (Figure 5G; Figure S9). Moreover, treatment with the Btk inhibitor ACP‐196 reduced the spread area and eliminated differences between WT and Rubicon^−/−^ platelets (Figure 5H).

**TABLE 2:** Ubiquitination sites of Btk in platelets. Identification of the ubiquitination site of Btk and analysis of the ubiquitination level of Btk in resting and Mn^2+^ stimulated platelets.

Protein accession	Position	Amino acid	KO_rest/WT_rest Ratio	KO_Mn^2+^/WT_Mn^2+^ Ratio	Protein description	Gene name	Score	Modified sequence
P35991	12	K	0.927914902	1.480767179	Tyrosine‐protein kinase BTK OS = Mus musculus OX = 10090 GN=Btk PE=1 SV=4	Btk	146.27	AAVILESIFLK(1)R

These data establish that Rubicon bidirectionally regulates platelet function through two distinct mechanisms: (1) direct inhibition of Btk in the GPVI pathway through RUN domain interaction, and (2) prevention of selective autophagic degradation of Btk to promote αIIbβ3 outside‐in signaling.

### 2.4 Platelet Rubicon Protects Against Cerebral Ischemia‐Reperfusion Injury and Hemorrhagic Transformation

**FIGURE 6:**
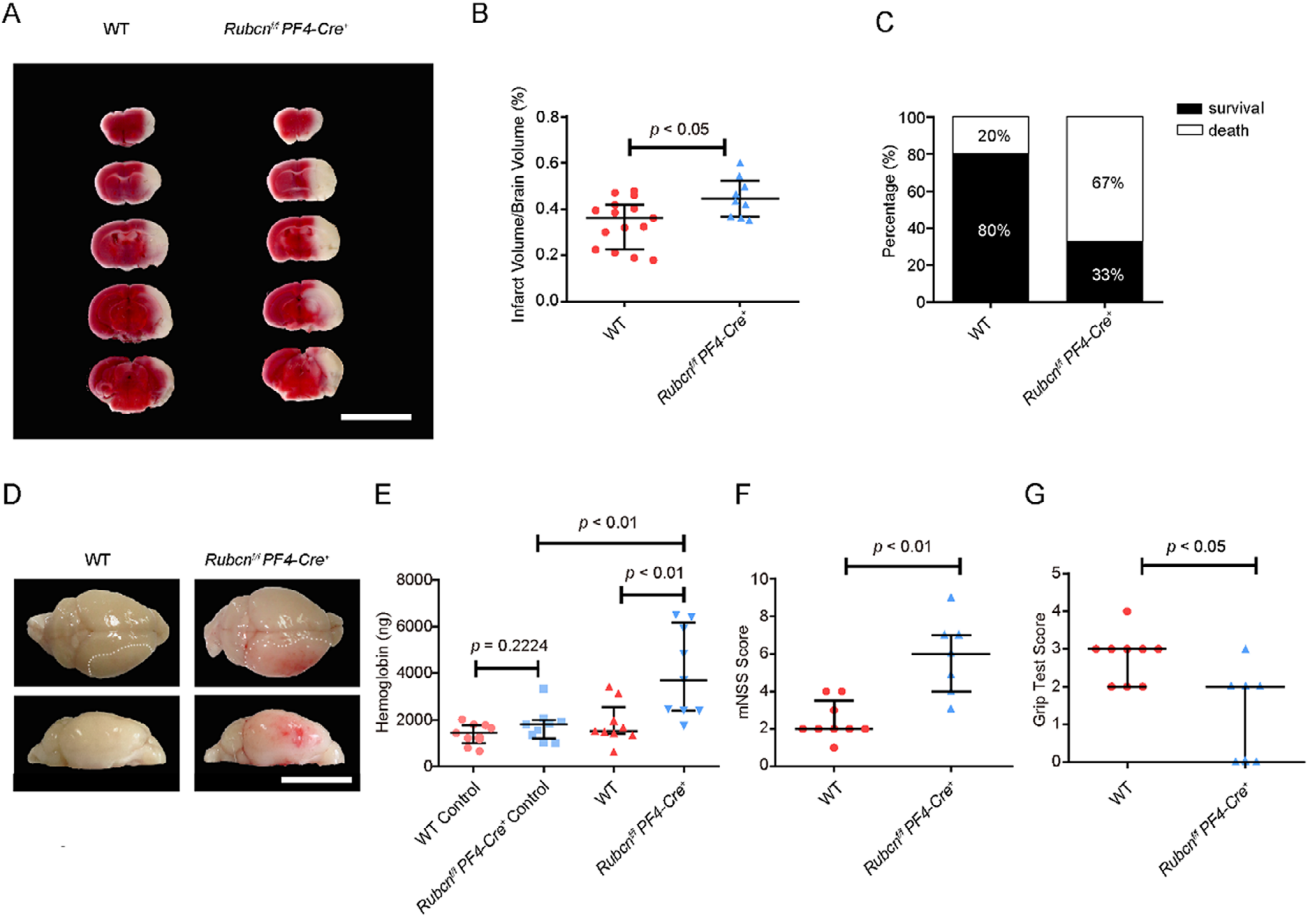
Platelet Rubicon protects against cerebral ischemia‐reperfusion injury and hemorrhagic transformation. (A) Representative images of 5 corresponding coronal sections from control and *Rubcn^f/f^ PF4‐Cre^+^* mice stained with TTC 24 h after tMCAO. Scale bar, 1 cm. (B) Brain infarct volumes are quantified by planimetric analysis 24 h after tMCAO in WT (●) and *Rubcn^f/f^ PF4‐Cre^+^* mice (▲). (C) Mortality rates 24 h after tMCAO. (D) Representative images of cerebral hemorrhage (top and dorsal views) post‐tMCAO. The area within the white dot represents the infarct area. (E) Quantification of cerebral hemorrhage by ELISA in control and stroke hemispheres. (F,G) Neurological deficit (mNSS) and grip test scores at day 1 post‐tMCAO. Statistical significance was evaluated with a two‐tailed Mann–Whitney *U* test; the data are shown as median with interquartile range.

Given the dual regulatory roles of Rubicon in GPVI and αIIbβ3 pathways and the pivotal roles of these two pathways in promoting cerebral infarction and preventing cerebral hemorrhage, respectively [3, 32], we investigated its pathophysiological significance in a transient middle cerebral artery occlusion (tMCAO) model of ischemic stroke (Figure 6A). In *Rubcn ^f/f^ Pf4‐Cre^+^* mice, cerebral infarct volumes were increased by more than 32% compared to controls (45 ± 2.8% vs. 34 ± 2.6%; Figure 6B), with significantly higher mortality rates (Figure 6C). Interestingly, consistent with our in vitro findings, platelets from *Rubcn ^f/f^ Pf4‐Cre^+^* mice that underwent tMCAO have significantly reduced total Btk levels, while an increased ratio of phospho‐Btk/total Btk when compared with platelets from WT mice with tMCAO (Figure S10). Strikingly, cerebral hemorrhage, measured by hemoglobin content in brain tissue, was markedly increased in Rubicon‐deficient mice (Figure 6D,E), coinciding with significantly worse neurological function (mNSS Score: 5.86 ± 0.77 vs. 2.44 ± 0.34) and motor performance (Grip Test Score: 1.29 ± 0.47 vs. 2.78 ± 0.22) (Figure 6F,G). Importantly, in a permanent MCAO model without reperfusion, no differences in infarct volumes were observed between genotypes (Figure S11), indicating that Rubicon's protective effects are specific to the reperfusion phase of ischemic injury. Then, the role of Rubicon in the secondary injury processes triggered by reperfusion was investigated. In addition to the finding that Rubicon^−/−^ platelets generated significantly higher levels of ROS than WT platelets upon collagen‐related peptide (CRP) stimulation (Figure S5C), flow cytometric analysis revealed that Rubicon^−/−^ platelets exhibit markedly enhanced intracellular Ca^2^^+^ mobilization following CRP stimulation (Figure S12A). Evans blue extravasation assays demonstrated significantly greater blood–brain barrier (BBB) permeability in *Rubcn^f/f^ PF4‐Cre^+^* mice following tMCAO (Figure S12B). Brain tissue analysis 24 h post‐tMCAO revealed significantly elevated IL‐6 levels in *Rubcn^f/f^ PF4‐Cre^+^* mice compared to WT controls (Figure S12C). Collectively, these results indicate that platelet Rubicon deficiency contributes to oxidative stress, calcium dysregulation, neuroinflammation, and blood–brain barrier (BBB) disruption, thereby exacerbating reperfusion injury.

Analysis of platelet Rubicon expression revealed significant down‐regulation 24 h after tMCAO compared to sham or pMCAO models (Figure S13A). This finding was corroborated in human samples. The demographic characteristics of patients and healthy controls (age, gender, risk factors, co‐medication) are shown in Table S2, while the major entry criteria are detailed in Table S3. Notably, platelets from stroke patients undergoing thrombectomy exhibited significantly reduced Rubicon expression compared to both non‐thrombectomy patients and healthy controls (Figure S13B). In vitro, Rubicon expression in platelets decreased upon collagen stimulation in a concentration and time‐dependent manner (Figure S13C,D), and this degradation was prevented by the proteasome inhibitor MG132 (Figure S14). These results indicate that Rubicon downregulation is intrinsically associated with platelet activation and ischemia‐reperfusion processes.

### 2.5 A Cell‐Permeable Rubicon Peptide Reduces Stroke Injury in a tMCAO Model

**FIGURE 7:**
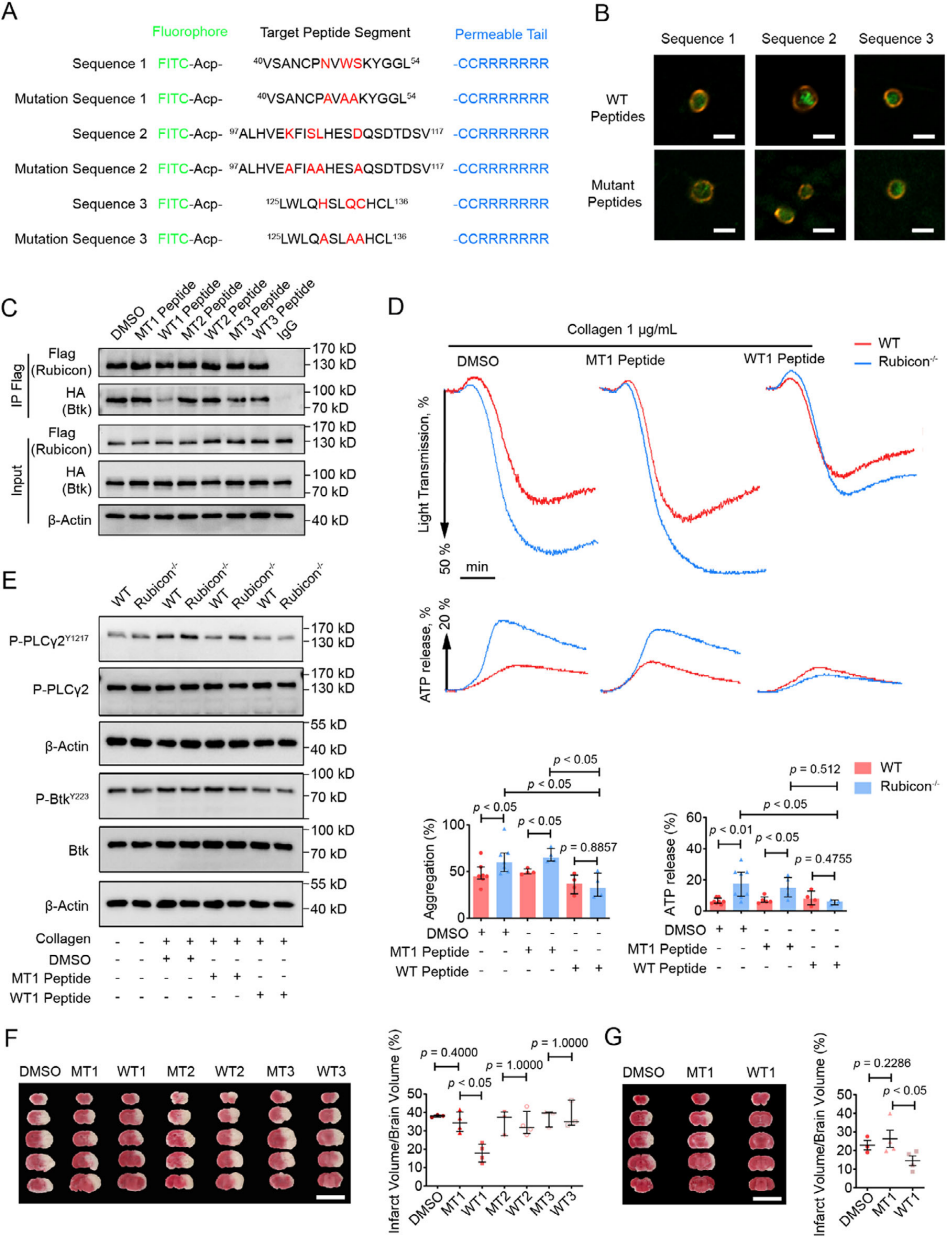
A cell‐permeable Rubicon peptide disrupts Rubicon‐Btk interaction and reduces stroke injury. (A) Schematic of designed cell‐permeable peptides: wild‐type and mutant sequences. Amino acid residues at the binding site are shown in red in the WT sequences and are replaced by alanine in the corresponding mutant sequences. (B) Fluorescence images showing uptake of FITC‐labeled peptides by platelets. Red, Tubulin. Scale bar, 2 µm. (C) Immunoprecipitation of FLAG‐Rubicon and HA‐Btk in HEK293T cells treated with DMSO, mutant, or wild‐type peptides. (D) Aggregation and ATP secretion in platelets treated with DMSO, WT1, or MT1 peptide and stimulated with collagen. Quantification of platelet aggregation (left) and ATP release (right). (E) Western blot analysis of phosphorylated PLCγ2 (Tyr^1217^) and Btk (Tyr^223^) in WT and Rubicon^−/−^ platelets stimulated with collagen in the presence of DMSO, MT1 peptides, or WT1 peptides. (F) Representative TTC‐stained brain sections and quantification of infarct volume in mice injected with DMSO or 2 mg·kg^−1^ peptides 5 min pre‐occlusion stained with TTC 24 h after tMCAO. Scale bar, 1 cm. (G) Representative TTC‐stained brain sections and quantification of infarct volume in mice injected with peptide or vehicle 85 min after occlusion (pre‐reperfusion). Statistical significance was evaluated with a two‐tailed Mann–Whitney *U* test; the data are shown as median with interquartile range.

Based on our findings that Rubicon expression decreases during ischemia‐reperfusion and that Rubicon deficiency exacerbates stroke outcomes, we hypothesized that supplementing Rubicon function might alleviate stroke injury. Knowledge‐based protein–protein docking employing HawkDock web‐server was first performed to identify the putative binding mode between the RUN domain of Rubicon and PH domain of Btk. Shown in Figure S15, the 3D structures of Rubicon‐Btk complexes were predicted by HawkDock, the best docked pose was predicted according to the Hawkdock score (−4391.54), and the free binding energy (−57.7 kcal/mol) was calculated by molecular mechanics‐generalized‐born surface area (MM‐GBSA, Tables S4 and S5) algorithm. The key residues for protein–protein interactions (PPIs) are provided in Table 3. We designed Rubicon peptides containing key‐binding residues and corresponding mutant peptides with those residues replaced by alanine (Figure 7A). A hepta‐Arg tag (RRRRRRR, R7) [33] was added to the C‐terminal end of the peptide for efficient cellular delivery, as confirmed by immunofluorescence (Figure 7B).

**TABLE 3:** Free binding energy from low to high of the top ten predicted amino acids in the best models.

Rank	Residue (Rec)	Binding free energy(kcal/mol)
1	Tyr‐132	−6.56
2	Ser‐49	−4.06
3	His‐128	−3.1
4	Trp‐48	−3.03
5	Asn‐46	−2.93
6	Ser‐104	−2.23
7	Val‐105	−2.21
8	Lys‐101	−2.19
9	Gln‐131	−1.57
10	Asp‐109	−1.15

Among these peptides, the WT1 peptide effectively disrupted Rubicon‐Btk binding in transfected HEK293T cells (Figure 7C) and significantly decreased collagen‐induced platelet aggregation and ATP release in WT platelets, eliminating differences between WT and Rubicon^−/−^ platelets (Figure 7D). Biochemically, the peptide reduced phosphorylation of PLCγ2 and Btk (Figure 7E), confirming its inhibitory effect on GPVI signaling.

To evaluate therapeutic potential, mice were injected with Rubicon peptides prior to tMCAO. Twenty‐four hours after reperfusion, infarct volumes were significantly reduced in mice treated with the WT1 peptide but not with DMSO or other peptides (Figure 7F). Remarkably, the peptide retained efficacy even when administered 85 min after occlusion onset (5 min before reperfusion) (Figure 7G), indicating a substantial therapeutic window that could be clinically relevant.

These results demonstrate that a peptide mimicking Rubicon's regulatory interaction with Btk effectively reduces ischemic injury in a stroke model, providing proof‐of‐concept for a novel therapeutic approach that targets the platelet‐specific mechanisms underlying cerebral ischemia‐reperfusion injury.

## 3 Discussion

**FIGURE 8:**
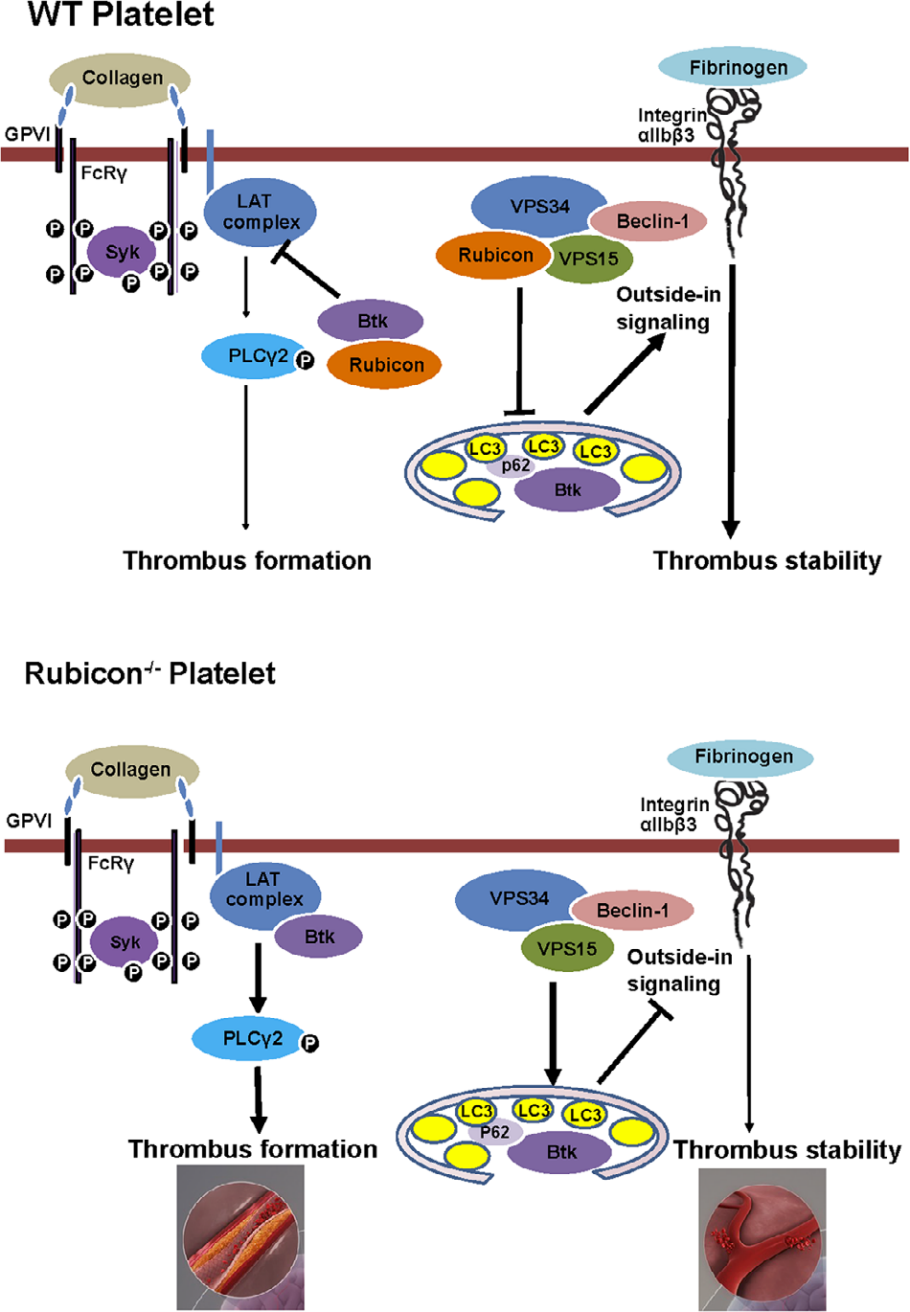
Schematic diagram of platelet Rubicon in regulating Platelet activation and thrombosis. In platelets, Rubicon acts as a molecular switch by dynamically exchanging binding partners to fine‐tune GPVI and integrin downstream signaling. Upon collagen‐induced GPVI activation, Rubicon dissociates from the class III PI3K complex and engages Btk, a critical downstream effector of GPVI. This interaction suppresses excessive Btk recruitment to the GPVI signaling cascade, thereby limiting signal amplification and preventing uncontrolled thrombus formation. Conversely, during integrin‐mediated signaling, Rubicon safeguards Btk stability by inhibiting selective autophagy. Integrin activation triggers pathways that promote autophagic degradation of signaling components, but Rubicon counteracts this process, preserving Btk levels to sustain integrin αIIbβ3‐dependent signaling and ensure thrombus stability. Genetic ablation of platelet Rubicon disrupts this dual regulatory mechanism. In Rubicon‐deficient platelets: Unrestricted Btk recruitment drives exaggerated GPVI responses, resulting in rapid and excessive thrombus formation. Loss of Rubicon‐mediated autophagy inhibition leads to unchecked Btk degradation, impairing integrin signaling and unstable thrombi.

Our study presents the first evidence that Rubicon serves as a critical protective factor in cerebral ischemia‐reperfusion injury (CIRI) through the differential regulation of platelet GPVI and integrin αIIbβ3 signaling pathways. Through its interaction with Btk, Rubicon inhibits GPVI‐mediated inside‐out signaling to prevent excessive platelet activation and reduce infarction, while simultaneously inhibiting autophagic degradation of Btk, which promotes integrin αIIbβ3‐mediated outside‐in signaling to enhance thrombus stability and prevent hemorrhage (Figure 8). This dual regulatory mechanism provides a molecular explanation for the previously observed paradoxical effects of GPVI versus αIIbβ3 inhibition in stroke models and opens new avenues for therapeutic intervention [3, 32].

Using megakaryocyte/platelet‐specific Rubicon‐deficient mice, we demonstrated that Rubicon deficiency significantly increases cerebral infarct volume and secondary hemorrhage during ischemia‐reperfusion injury. This phenotype directly corresponds to Rubicon's functions in platelet signaling: specific inhibition of the GPVI pathway and enhancement of integrin αIIbβ3 outside‐in signaling. Previous studies have established the role of GPVI in promoting cerebral infarction and the requirement of αIIbβ3 in preventing secondary bleeding [3]; our findings now indicate that these seemingly disparate functions are intrinsically unified through Rubicon regulation. The dual mechanism of Rubicon action provides a potential solution to a major clinical challenge in antiplatelet therapy. Although evidence suggests a minimal risk in healthy individuals [34], the bleeding risk associated with GPVI inhibition remains a significant concern, as demonstrated in studies where anti‐GPVI treatment was combined with aspirin therapy, which severely compromised hemostasis [35]. Rubicon achieves a balanced regulatory effect by inhibiting GPVI‐mediated thrombosis while simultaneously strengthening αIIbβ3‐mediated thrombus stability, potentially mitigating bleeding risk. This bidirectional regulatory mechanism may potentially offer crucial safety benefits. However, as the Rubicon‐derived WT1 peptide indeed reduced infarct volume, its effect on intracerebral hemorrhage warrants further investigation.

At the molecular level, we discovered that Rubicon constitutively binds to the VPS34 complex in resting platelets but dissociates upon GPVI activation. While this interaction aligns with Rubicon's established role as a negative regulator of VPS34 [30, 36], our findings reveal a more complex picture. Rubicon‐regulated platelet activation differs from VPS34‐mediated effects [12] in several important ways: Rubicon selectively regulates GPVI‐mediated but not GPCR‐mediated activation, and although Rubicon influences PI(3)P production and ROS generation, eliminating ROS did not abolish the differences in collagen‐induced aggregation between Rubicon‐deficient and wild‐type platelets. These observations indicate that Rubicon regulates GPVI signaling through mechanisms beyond VPS34‐ROS generation.

The identification of Btk as a Rubicon‐interacting partner provides a mechanistic explanation for its effects on GPVI signaling. In agreement with the previously reported importance of the PH domain in membrane targeting [37] and the kinase activity of Btk [38, 39, 40, 41, 42], our data demonstrate that the interaction between RUN domain of Rubicon and PH domain of Btk is crucial for Rubicon's inhibitory function. Our results suggest a model in which Rubicon dynamically switches its binding partner from VPS34 to Btk upon GPVI activation. Importantly, this Btk inhibition appears to be specific to the GPVI pathway, as no interaction or functional differences were observed with thrombin stimulation. Despite the lack of separate evaluation of the Rubicon‐derived peptides in the bleeding transformation of CIRI, our findings demonstrate their bidirectional fine‐tuning effects in platelet activation. This suggests that targeting Rubicon may offer a superior safety strategy to the global BTK inhibitors (e.g., Ibrutinib), which carry inherent bleeding risks [43]. Moreover, clinical trials conducted on GPVI inhibitors such as Revacept [44] and glenzocimab (ACT017) [32] have established favorable bleeding safety profiles, thus validating the concept that lesion‐directed or pathway‐selective antiplatelet strategies can achieve antithrombotic efficacy without compromising hemostasis. Our Rubicon‐targeting approach aligns with this paradigm but operates through a distinct mechanism involving autophagy‐platelet function crosstalk, which may offer complementary or synergistic therapeutic potential in CIRI. Future studies addressing the pharmacokinetics, toxicity, immunogenicity, and efficacy on bleeding prevention of the Rubicon‐mimetic peptides are warranted to further their clinical translation.

Our study also provides novel insights into how autophagy integrates with platelet activation. We demonstrate that selective autophagy is specifically embedded in platelet integrin αIIbβ3 outside‐in signaling and that Rubicon inhibits this process. Our results extend previous observations that integrin activation can induce autophagy and identify Btk as a cargo selectively degraded by αIIbβ3‐induced autophagy. By inhibiting the autophagic degradation of Btk, Rubicon promotes αIIbβ3 outside‐in signaling and thrombus stability. This establishes a unified mechanism where Rubicon differentially regulates GPVI and αIIbβ3 pathways through distinct targeting of the same molecule, Btk.

The downregulation of Rubicon expression following collagen stimulation or ischemia‐reperfusion further highlights its importance as a negative regulator of platelet activation. This reduction in expression not only confirms Rubicon's role as a brake on GPVI‐mediated activation but also provides a rationale for therapeutic strategies aimed at restoring Rubicon function. Our peptide mimicking the Rubicon‐Btk interaction effectively reduced platelet aggregation in vitro and decreased infarct volume in the tMCAO mouse model, demonstrating proof‐of‐concept for this approach. A particularly promising finding is the effectiveness of our Rubicon‐derived peptide when administered post‐ischemia but pre‐reperfusion. This therapeutic window of at least 85 min post‐occlusion distinguishes our approach from purely preventive strategies and enhances its potential clinical relevance, especially given the unpredictability of ischemic events in real‐world settings.

This study has several limitations. While the switch of interacting partner and subsequent function of Rubicon is clearly GPVI‐dependent, it is not clear how GPVI activation led to these alterations. Moreover, while we focused on the RUN domain of Rubicon, the potential roles of other domains (e.g., FYVE, CCD, and S‐R [17]) in platelet activation were not thoroughly investigated. Furthermore, the patient cohort size in this study is relatively small. Future research focusing on expanding the clinical sample size is warranted to further elucidate the mechanism and therapeutic value of Rubicon in ischemic stroke.

In conclusion, our study provides compelling evidence that platelet Rubicon plays a protective role in CIRI through both autophagic and non‐autophagic mechanisms that differentially regulate the key signaling molecule Btk. As Rubicon degradation is intrinsically associated with platelet activation, restoring its molecular function represents a promising therapeutic strategy to mitigate stroke progression by simultaneously addressing both excessive thrombosis and risk of hemorrhagic transformation.

## 4 Materials and Methods

### 4.1 Materials

The following antibodies were used: anti‐VPS15 (Novus Biologicals, NBP1‐30463); anti‐PIK3C3 (Cell Signaling, 4263S); anti‐BECN1 (Cell Signaling, 3738S); anti‐LC3 (clone 2G6) (nano Tools, 0260); anti‐Tubulin α (sigma, T5168); anti‐p‐Akt ^S473^ (Cell Signaling,4060S); anti‐p‐Akt ^T308^ (Cell Signaling, 13038S); anti‐p‐ERK (Cell Signaling, 9101S); anti‐p‐p40phox (Santa Cruz, sc33403); anti‐p‐integrin β3 (Santa Cruz, sc365679), anti‐p‐integrin β3^T747^ (Santa Cruz, sc101707), anti‐p‐integrin β3^T759^ (Santa Cruz, sc20235); FITC‐conjugated anti‐mouse CD41 monoclonal antibody (mAb) (BD Biosciences, MWReg30, 558040); FITC‐conjugated anti‐mouse CD42b mAb (Emfret Analytics GmbH & Co. KG, Xia.B2, M043‐0); FITC‐conjugated anti‐mouse GPVI mAb (Emfret Analytics, JAQ1, M011‐1); anti‐Rubicon antibodies (Cell Signaling, D9F7, 3412; GeneTax, GTX129096); anti‐Btk antibody(Cell Signaling, D3H5, 8547); anti‐p‐Btk^Y223^ antibody (Cell Signaling, D1D2Z, 87457); anti‐PLCγ2 antibody (Cell Signaling, 3872); anti‐p‐PLCγ2^Y1217^ antibody (Cell Signaling, 3871T); anti‐p‐Lyn^Y507^ antibody (Cell Signaling, 2731T); anti‐fyn antibody (Abcam, 2A10, ab119855); anti‐p‐Fyn^Y530^ antibody (Abcam, ab182661); anti‐p62 antibody (Cell Signaling, D6M5X, 23214); anti‐LC3 antibody (Sigma Aldrich, L7543 and L8919); anti‐LAMP2 antibody (Proteintech, 66301). Alexa Fluor 488‐ (A11029) and 546‐ (A11035) tagged secondary antibodies for immunostaining, and FLUO‐4 AM were from Invitrogen. F2/thrombin (Sigma, T6884), 3‐methyladenine (M9281), H2DCFDA (D6665), mepacrine (Q3251), and ADP (A3515) were from Sigma Aldrich. Collagen (385) and luciferase (395) were from Chrono‐Log Corporation. U46619 (538944) was from Millipore. LysoSensor Green DND‐189 (40767ES) was from Yeasen Biotechnology (Shanghai) Co., Ltd. Evans blue (BS177‐1 g) was from Biosharp. A serotonin ELISA kit (ADI‐900‐175) was from Enzo Biochem, Inc. Mouse hemoglobin (Hb) ELISA kit (JL20632‐96T) was from Shanghai Jianglai Industrial Limited by Share Ltd. Interleukin ‐6 ELISA kit (H007‐1) was from Nanjing Jiancheng Bioengineering Institute. A PI(3)P Mass ELISA Kit (K‐3300) was from Echelon Biosciences Inc.

### 4.2 Human Studies

All studies involving human participants complied with the ethical standards of the Declaration of Helsinki and were approved by the Ethics Committee of Lishui Central Hospital (IRB Approval No.: 2022‐04). Participants provided written informed consent, and exclusion criteria included recent anticoagulant use or bleeding disorders (Table S3).

### 4.3 Animal Studies

*Rubcn^flox/flox^* mice were generated by flank the exon 4 of the *Rubcn* gene with LoxP sites. Megakaryocyte/platelet‐specific Rubicon KO mice were generated by crossing *PF4‐Cre^+^* mice (Model Animal Research Center, Nanjing University, China) with *Rubcn^flox/flox^* mice. All mice used in the experiments were 8–14 weeks old, with weight and sex matched across groups. All animal protocols were approved by Zhejiang University Laboratory Animal Welfare and Ethics Committee (Protocol # ZJU20240170).

### 4.4 Preparation of Washed Mice Platelets

As previously described [45], whole blood was collected from the inferior vena cava into ACD (1/9 v/v; 75 mM sodium citrate, 39 mM citric acid and 135 mM dextrose, pH 6.5) and was diluted 1:3 with modified Tyrode's buffer (20 mM HEPES, 137 mM NaCl, 13.8 mM NaHCO_3_, 2.5 mM KCl, 0.36 mM NaH_2_PO_4_, 5.5 mM glucose, pH 7.4). To isolate platelets, the diluted whole blood was first centrifuged at 180 × g for 10 min at room temperature to obtain platelet‐rich plasma (PRP). The PRP was then subjected to a second centrifugation at 800 × g for 10 min. The platelet pellet was resuspended in modified Tyrode's buffer for further use.

### 4.5 Preparation of Washed Human Platelets

Platelets were collected from cerebral ischemic stroke patients (with or without thrombectomy) at Lishui Central Hospital. Blood was collected without stasis into siliconized vacutainers containing 3.8% sodium citrate at a ratio of 1:9 v/v. Platelets were subsequently isolated, washed, and resuspended following previously established protocols [46]. Samples were taken at admission or 24 h post‐thrombectomy, and platelet lysates were stored at −80°C until use.

### 4.6 Middle Cerebral Artery Occlusion (MCAO) Model

Transient middle cerebral artery occlusion (tMCAO) was performed as described [47, 48, 49]. Eight‐week‐old littermates of WT or *Rubcn^f/f^ PF4‐Cre^+^* mice with C57BL/6J background (20–25 g) were anesthetized, and a midline cervical incision was made.

The common and external carotid arteries were ligated, and a blunt‐tipped 6‐0 nylon filament was inserted into the right common carotid artery and advanced ∼10 mm to occlude the middle cerebral artery (MCA) origin. The filament was left in place for 90 min and then removed for reperfusion. For permanent MCA occlusion (pMCAO), the filament remained in place for 24 h. Neurological deficits were assessed using the modified neurological severity score (mNSS, 0‐14), and motor coordination was evaluated with a grip test (score 0‐5) [50]. Cell‐permeable peptides (2 mg/kg) were intravenously injected either before tMCAO model establishment or 5 min prior to reperfusion during the 85‐min ischemic period.

### 4.7 Quantification of Cerebral Hemorrhage and IL‐6 Level in Brain Tissues

Following a previously described method [51], mice were perfused with phosphate‐buffered saline after MCAO. Brain tissue was homogenized and centrifuged at 13,000 g for 30 min, and the levels of hemoglobin and IL‐6 in the supernatant were measured using a mouse hemoglobin ELISA kit and IL‐6 ELISA kit.

### 4.8 Evans Blue Staining

Evans blue extravasation was measured as previously described [52]. Mice were administered 0.5% Evans blue dye via tail vein injection at a dose of 2 mL/kg. After 30 min, the mice were euthanized and perfused with ice‐cold phosphate‐buffered saline through the left ventricle until colorless fluid outflowed from the right atrium. Then, the ipsilateral and contralateral hemispheres were collected after decapitation. Each brain sample was placed in a 1.5 mL centrifuge tube, followed by the addition of 1 mL of 50% trichloroacetic (diluted with PBS). The tissue was rapidly homogenized using a tissue homogenizer. Evans blue was extracted at 55°C overnight and centrifuged at 13,000 g for 30 min. The resulting supernatant was collected and diluted fourfold with absolute ethanol. Absorbance at 620 nm was measured using a spectrophotometer to determine the optical density (OD). The Evans blue concentration in the samples was then calculated based on the standard curve.

### 4.9 The Synthesis of Peptides

Rubicon‐derived and scrambled peptides were synthesized according to a previously described method [53]. using FMOC Solid Phase Peptide Synthesis with an additional C‐terminal cysteine. A cell‐penetrating sequence (CRRRRRRR) was conjugated via a disulfide bond, and FITC was attached to the N‐terminus. Peptides were purified by HPLC and confirmed by ESI‐MS (SHIMADZU LCMS‐2020, Kyoto, Japan).

### 4.10 Tail‐Bleeding Time Assay

Mice were anesthetized with 4% isoflurane for induction and maintained with 1.5% isoflurane in 100% oxygen (2–3 L/min). In comparison with other anesthetics such as sevoflurane, isoflurane anesthesia provides a stable physiological [54] condition without affecting bleeding [55, 56] and therefore is a reliable anesthesia method for the tail bleeding assay. The tails of anesthetized mice were cut 0.3 cm from the tip and immersed in 37°C saline. Bleeding time was recorded as the duration until bleeding ceased (no flow for 1 min). Assays were terminated at 600 s if bleeding persisted.

### 4.11 Intravital Microscopy of FeCl3‐Injured Thrombus Formation in Mouse Mesenteric Arteriole

Mepacrine (quinacrine dihydrochloride)‐labeled fluorescent platelets (10^8^) were injected via the tail vein. The mesentery was exposed and placed on a translucent stage for fluorescence microscopy. Mesenteric arteriole injury (60–100 µm diameter) was induced with 10% FeCl_3_, and arterioles were monitored for 40 min or until occlusion (blood flow cessation for >1 min).

### 4.12 Thrombus Formation Under Flow Conditions in Vitro

As previously described [45], thrombus formation in vitro was assessed using the Bioflux‐200 system (Fluxion, CA, USA). Microfluidic plates were coated with collagen (50 µg/mL), and freshly isolated mouse whole blood was anticoagulated with heparin (7.5 U/mL) and labeled with mepacrine (100 µM). Blood was perfused over the collagen‐coated surface for 5 min at specified shear rates to visualize platelet adhesion and thrombus formation. Adherent platelets were imaged using an inverted fluorescence microscope (×20/0.4 objective) and a Nikon DS‐Qi1‐U3 CCD camera. The platelet‐covered area was quantified using Bioflux software (Fluxion, San Francisco, CA, USA).

### 4.13 Hematologic Analysis

Complete blood counts and hematocrits were conducted by Animal hospital affiliated to Zhejiang University with ProCyteDx hematology analyzer (IDEXX Laboratories, Westbrook, ME, USA).

### 4.14 Platelet Aggregation and ATP Secretion

Platelet aggregation and ATP secretion were measured using a lumi‐aggregometer at 37°C with stirring (1200 rpm). Washed platelets (2 × 10^8^/mL) in modified Tyrode buffer were stimulated with thrombin, collagen, U46619, or ADP (with fibrinogen). ATP release was assessed using luciferin/luciferase reagent. Inhibitors were pre‐incubated with platelets for 5 min before stimulation.

### 4.15 Serotonin Content and Release Assay

Washed platelets (1.5–3 × 10^8^) were resuspended in 0.2 mL distilled water and stored at −70°C until analysis. Serotonin levels were measured using a serotonin ELISA kit following the manufacturer's instructions. Experiments were repeated at least five times, and results are presented as mean ± SEM. Statistical significance was determined using the Mann–Whitney *U* test.

### 4.16 TXB_2_ Content and Release Assay

Washed platelets (2 × 10^8^/mL) were stimulated with 1 µg/mL collagen for 5 min and then lysed with Cell Signaling Technology 10 × lysis buffer. Allow the cell lysis solution to stand for 30 min on ice, and then centrifuge at 13,000 rpm for 10 min. TXB_2_ levels in the supernatant were measured using a TXB_2_ ELISA kit following the manufacturer's instructions. Experiments were repeated at least five times, and results are presented as mean ± SEM. Statistical significance was determined using the Mann–Whitney *U* test.

### 4.17 Platelet Spreading on Fibrinogen

Coverslips (18 × 18 mm) were coated overnight at 4°C with 20 µg/mL fibrinogen in 0.1 M NaHCO_3_ (pH 8.3). Washed platelets (2 × 10^7^/mL, 200 µL) were spread on the fibrinogen‐coated surface at 37°C for 60 min. After rinsing with PBS, platelets were fixed, permeabilized, and stained with phalloidin. Adherent platelets were imaged using a Nikon Ti–S inverted fluorescence microscope and DS‐Qi1‐U3 camera. Platelet‐covered areas were quantified with NIS‐D software (Nikon, Tokyo, Japan).

### 4.18 Clot Retraction

Murine platelets were resuspended in citrated human platelet‐depleted plasma at 4 × 10^8^/mL. Clot retraction was initiated by adding thrombin (0.4 U/mL) and monitored at room temperature, with photographs taken at specified intervals.

### 4.19 Flow Cytometric Analysis

WT or Rubicon^−/−^ platelets (10^6^) were labeled with specific antibodies (anti‐CD41, CD42b, and GPVI mAbs) for 10 min at room temperature. After agonist stimulation for 10 min, reactions were stopped with 1% formaldehyde. Samples were analyzed using an EPICSXL flow cytometer (Beckman Coulter, Fullerton, CA, USA).

### 4.20 Intracellular Ca^2+^ Flux Measurement

The platelet count was adjusted to 10^8^/mL with Tyrode's buffer. Platelets were incubated with the calcium‐sensitive fluorophore Fluo‐4 AM (3 µm; Invitrogen) for 30 min at 37°C in the dark. Fluo‐4‐loaded platelets were stimulated with CRP (1 µg/mL) in the presence or absence of Ca^2+^ (2 mM CaCl_2_). Fluorescence was detected by flow cytometry (Beckman Coulter, Fullerton, CA, USA). For real‐time flow cytometry, events were collected at a slow flow rate for 5 min after stimulation.

### 4.21 Electron Microscopy

Washed wild‐type (WT) or Rubicon^−/−^ platelets were fixed with 2.5% glutaraldehyde, processed through standard staining and dehydration protocols, and embedded. Thin sections were stained with uranyl acetate and lead citrate. Samples were examined using a Tecnai 10 transmission electron microscope at 80 kV, with images captured using an ES500W (782) camera and Digital Micrograph software (Gatan, Pleasanton, CA, USA).

### 4.22 Immunoblotting

Washed platelets (2×10^8^/mL) were stimulated in a Chrono‐Log aggregometer, and reactions were stopped with 4× SDS lysis buffer (4 mM NaVO3, 4 mM NaF, 4% (g/vol) SDS, and 500 mM DTT, pH 6.8) containing protease and phosphatase inhibitors. For spreading assays, platelets (4×10^7^/mL) were spread on fibrinogen for 60 min and lysed with 1× RIPA buffer. Proteins were separated by SDS‐PAGE gels, transferred to PVDF membranes, and probed with primary antibodies followed by HRP‐conjugated secondary antibodies. Proteins were visualized using enhanced chemiluminescence and imaged on a Bio‐Rad ChemiDoc MP system (Bio‐Rad, Hercules, CA, USA).

### 4.23 Immunoprecipitation Assay and LC‐MS/MS Mass Spectrometry

Stimulated platelets were lysed with 5× lysis buffer (250 mM Tris pH 7.4, 750 mM NaCl, 5 mM NaF, 5 mM NaVO_3_, 5% NP40, 50 mM MgCl_2_) containing protease and phosphatase inhibitors at 4°C. Lysates were immunoprecipitated using anti‐Rubicon antibodies overnight at 4°C, followed by protein A/G‐agarose incubation for 2–4 h. After washing, bound proteins were eluted with 2 × loading buffer and analyzed by immunoblotting. SDS‐PAGE gels were also subjected to mass spectrometry analysis.

### 4.24 Confocal Microscopy

Platelets were spread on Poly‐L‐Lysine or fibrinogen‐coated coverslips for 60 min, then fixed and permeabilized. Samples were incubated with anti‐p62 and anti‐Btk primary antibodies overnight, followed by Alexa Fluor 488‐ and 546‐tagged secondary antibodies for 2 h. Images were captured using a Zeiss LSM 800 confocal microscope with a 100×/1.49 NA oil immersion lens (0.5 µm optical slice thickness).

### 4.25 PI(3)P Mass Assay

Collect platelets into a 15 mL centrifuge tube, pellet by centrifugation, and remove the supernatant. Add 5 mL ice‐cold 0.5 M TCA, vortex, and incubate on ice for 5 min. Centrifuge at 3000 RPM (∼900‐1000 RCF) for 7 min at 4°C, discard the supernatant, and wash the pellet twice with 3 mL of 5% TCA/1 mM EDTA, vortexing for 30 s and centrifuging at 3000 RPM for 5 min each time. Resuspend the pellet in 3 mL MeOH: CHCl_3_ (2:1), vortex for 10 min at room temperature, and centrifuge at 3000 RPM for 5 min. Discard the supernatant and repeat the neutral lipid extraction once. Add 2.25 mL MeOH: CHCl_3_:12 N HCl (80:40:1), vortex for 25 min at room temperature, and centrifuge at 3000 RPM for 5 min. Transfer the supernatant to a new tube, add 0.75 mL CHCl_3_ and 1.35 mL 0.1 N HCl, vortex for 30 s, and centrifuge again to separate phases. Collect the organic (lower) phase using a positive displacement pipette, avoiding debris, and dry in a vacuum dryer (45–60 min). PI(3)P extraction samples were rehydrated and detected by PI(3)P Mass ELISA Kit.

### 4.26 ROS Production Measurement

Intracellular ROS was measured as previously described [12]. Washed platelets (1×10^8^/mL) were incubated with fluorogenic probe 2′,7′‐dichlorofluorescein diacetate (H_2_DCF‐DA, 50 µM) for 15 min at 37°C in the dark, then stimulated with collagen‐related peptide (CRP) for 5 min. Samples were diluted 10‐fold in Tyrode's buffer containing H_2_DCF‐DA (50 µm) and immediately analyzed by flow cytometry.

### 4.27 Molecular Docking of RUN Domain of Rubicon with PH Domain of Btk

The binding interaction between Rubicon and Btk was modeled using two protein–protein docking web servers: HawkDock (http://cadd.zju.edu.cn/hawkdock/) and ZDOCK (http:// https://zdock.wenglab.org/). This approach was chosen to achieve the most likely native conformation based on binding domain information from co‐immunoprecipitation studies. MM/GBSA free energy decomposition analysis was performed on a multipurpose platform. Multiple servers were used to obtain a consensus binding mode, as results can vary between different docking algorithms.

### 4.28 HEK293T Cell Culture, Transfection, and Peptide Treatment

HEK293T cells were cultured in DMEM supplemented with 10% FBS and antibiotics and transfected with plasmids expressing HA‐Btk together with either Flag‐tagged Rubicon or its truncated fragments as previously described [25]. After transfection, 2 × 10^6^ cells were collected and incubated with DMSO or 60 µm peptides for 5 min, then lysed with 1× lysis buffer. Lysates were immunoprecipitated overnight at 4°C with anti‐Flag antibodies, followed by protein A/G‐agarose incubation for 2–4 h. Bound proteins were eluted with 2 × loading buffer and analyzed by immunoblotting.

### 4.29 Statistical Analysis

Dot plots are presented as median with interquartile range or mean ± SEM. The results are shown as median with interquartile range. Data normality was assessed using the *Shapiro*–*Wilk* test, and the choice between parametric (Student's *t‐*test) and non‐parametric (Mann–Whitney *U* test) tests was based on this assessment. For comparisons involving more than two groups or variables, two‐way ANOVA with Bonferroni post hoc analysis was applied. Statistical significance was set at *p* <.05. Analyses were performed using GraphPad Prism 10 (GraphPad Software, San Diego, CA, USA).

## Author Contributions

X. Chen, J. Li, Y. Liu, L. Li, X. Deng, and X. Jiang performed the experiments and analyzed the data. X. Chen and J. Li wrote and revised the manuscript. Y. Sheng and X. Zhu contributed intellectually and revised the manuscript. W. Li performed the experiments. Xueli Cai, Qiming Sun, and Hu Hu designed the research and wrote and revised the manuscript.

## Funding

This study was supported by grants from the National Natural Science Foundation of China (U23A20416, 82070138), Zhejiang Provincial Natural Science Foundation (LZ23H080001), and the discipline construction funding of Innovation Institute of Basic Medical Sciences, Zhejiang University.

## Conflicts of Interest

The authors declare no conflicts of interest.

## Supporting information




**Supporting File 1**: advs73738‐sup‐0001‐SuppMat.docx.

**Supporting File 2**: advs73738‐sup‐0002‐VideoS1.mp4.

**Supporting File 3**: advs73738‐sup‐0003‐VideoS2.mp4.

**Supporting File 4**: advs73738‐sup‐0004‐VideoS3.mp4.

**Supporting File 5**: advs73738‐sup‐0005‐VideoS4.mp4.

**Supporting File 6**: advs73738‐sup‐0006‐VideoS5.mp4.

## Data Availability

The data that support the findings of this study are available from the corresponding author upon reasonable request.
