# Supplementary material for: Platelet Rubicon Bidirectional Regulation of GPVI and Integrin αIIbβ3 Signaling Mitigates Stroke Infarction Without Compromising Hemostasis
Source: Adv Sci (Weinh). 2026 Jan 21;13(16):e07509. doi: 10.1002/advs.202507509 (PMC13042973; doi:10.1002/advs.202507509)
Supplement: Supplementary file 1 — Supporting File 1: advs73738‐sup‐0001‐SuppMat.docx. [file ADVS-13-e07509-s006.docx]

**Supplementary Material**

**Platelet Rubicon Bidirectional Regulation of GPVI and Integrin αIIbβ3 Signaling Mitigates Stroke Infarction Without Compromising Hemostasis**

**Running title:** Platelet Rubicon Attenuates Ischemic Stroke Injury

Xiaoyan Chen^1,3#^, Jingke Li^1,3#^, Yangyang Liu^5^, Li Li^1,3^, Xin Deng^1,3^, Yilin Sheng^1,3^, Xianyu Zhu^1,3^, Xiao Jiang^6,7^, Wei Li^4^, Xueli Cai^4*^, Qiming Sun^2*^, Hu Hu^1,3*^.

^1^Department of Pathology and Pathophysiology and Bone Marrow Transplantation Center of the First Affiliated Hospital, Zhejiang University School of Medicine, Hangzhou, China;

^2^Department of Biochemistry and Molecular Biology, Zhejiang University School of Medicine, Hangzhou, China;

^3^Institute of Hematology, Zhejiang University & Zhejiang Engineering Laboratory for Stem Cell and Immunotherapy, Hangzhou, China;

^4^Lishui Central Hospital, the Fifth Affiliated Hospital of Wenzhou Medical University, Lishui Hospital of Zhejiang University, Lishui, China.

^5^Department of Cardiology, The First Affiliated Hospital of Zhengzhou University, Zhengzhou 450052, China;

^6^State Key Laboratory of Transvascular Implantation Devices, Department of Cardiology of The Second Affiliated Hospital, School of Medicine, Zhejiang University, Hangzhou, 310009, China;

^7^Heart Regeneration and Repair Key Laboratory of Zhejiang province

^#^These authors contributed equally to this study.

Address for correspondence:

Hu Hu, Department of Pathology and Pathophysiology and Bone Marrow Transplantation Center of the First Affiliated Hospital, Zhejiang University School of Medicine, Hangzhou 310058, P.R. China. Tel: +86-571-88208517. Fax: +86-571-88208197. E-mail: huhu@zju.edu.cn

Sun Qiming, Department of Biochemistry, and Department of Cardiology of Second Affiliated Hospital, Zhejiang University School of Medicine, Hangzhou 310058, P. R. China. Tel: +86-571-88208505. E-mail: qmsun@zju.edu.cn

Cai Xueli, Lishui Central Hospital, the Fifth Affiliated Hospital of Wenzhou Medical University, Lishui Hospital of Zhejiang University, Lishui, 323000, P. R. China. E-mail: xueli_cai_official@126.com

**
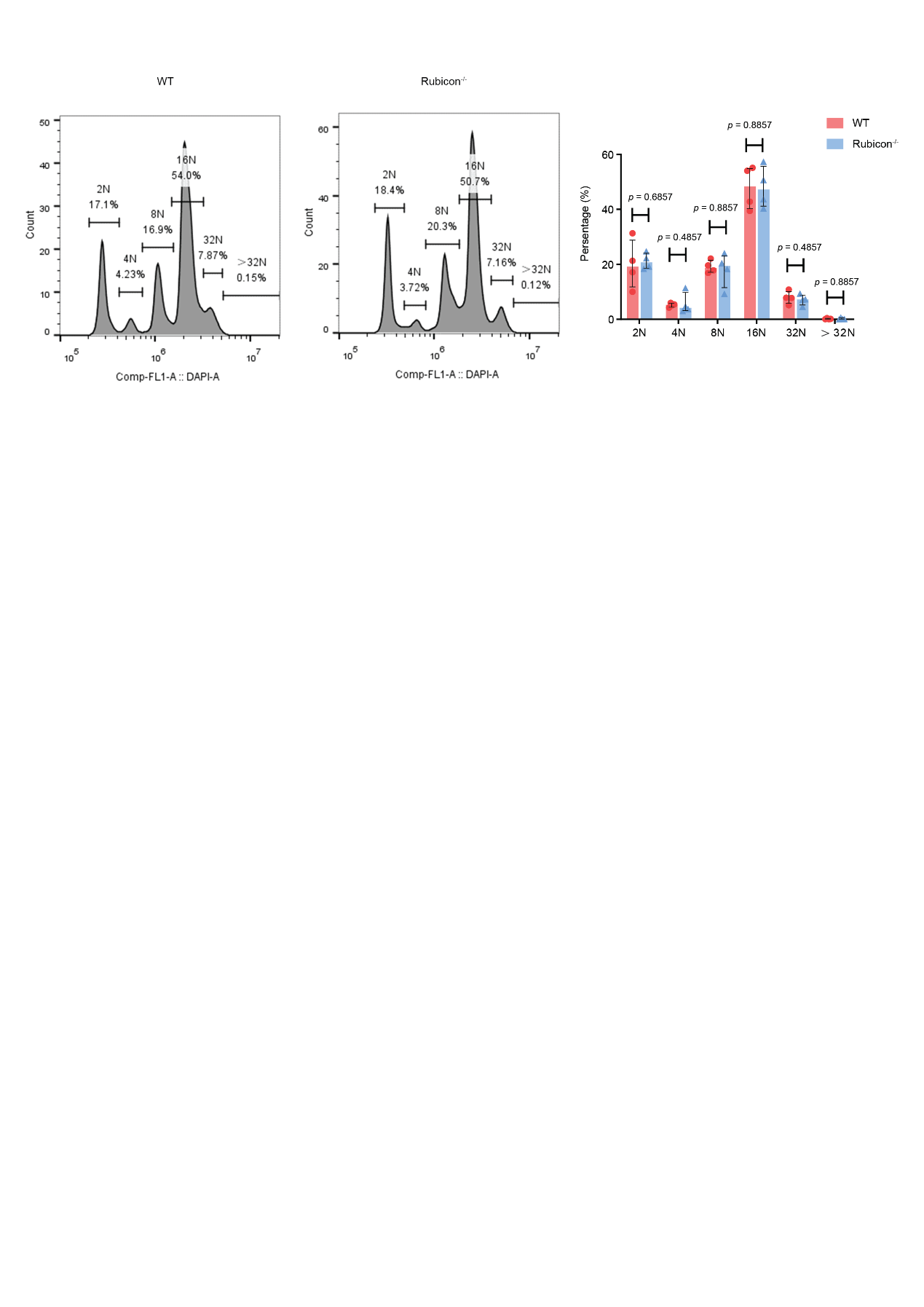
Figure S1. Deficiency of Platelet Rubicon did not affect the megakaryocytes ploidy distribution.** Ploidy analysis in megakaryocytes from WT and *Rubcn^f/f^ PF4-Cre^+^* mice, as determined by flow cytometry. Results are expressed as cell percentage, (*n* = 4). Statistical significance was evaluated with 2-tailed Mann-Whitney *U* test, the data are shown as median with interquartile range.


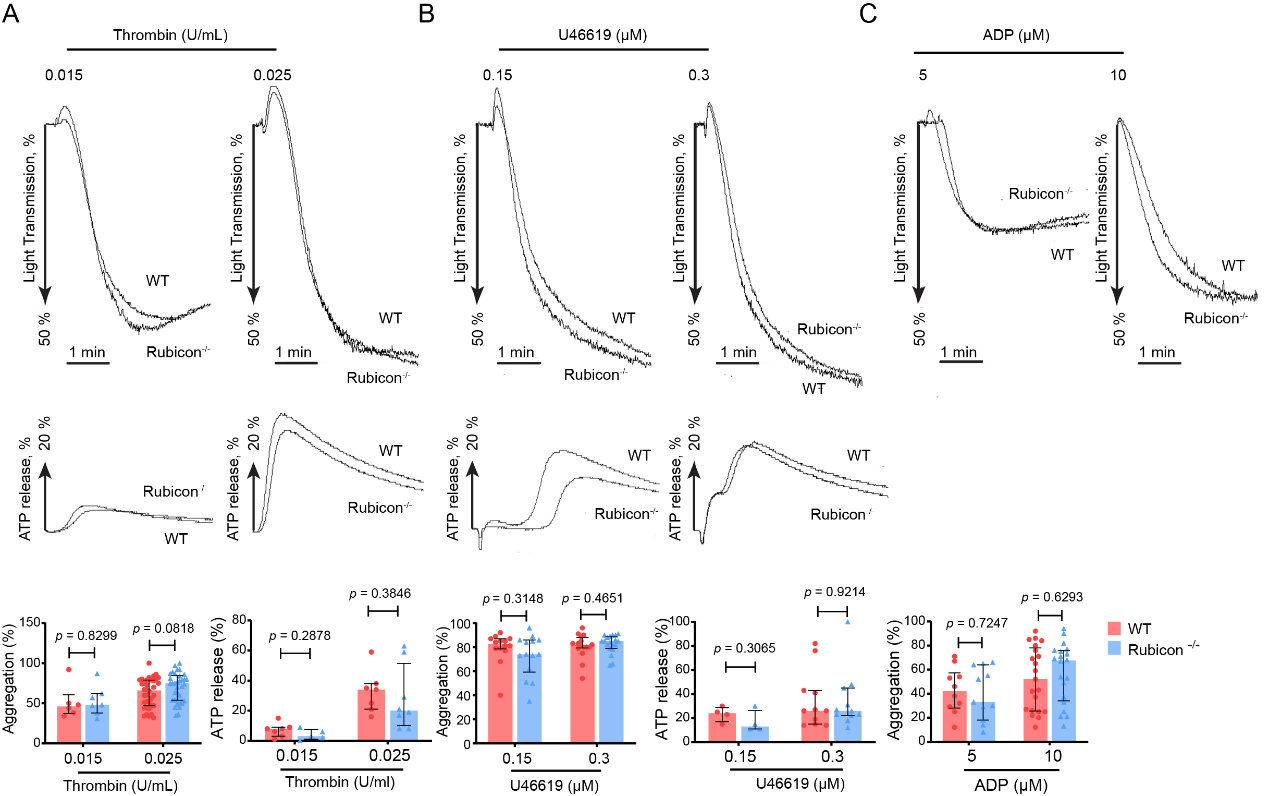
**Figure S2. Rubicon^-/-^ platelets aggregate normally in response to thrombin, U46619 and ADP.** (A) Washed platelets (2×10^8^ mL^-1^) were stimulated with indicated concentration of thrombin. Aggregation and ATP secretion (with luciferase) was assessed with a Chrono-log lumi-aggregometer under stirring at 1200 rpm. Percentage of platelet aggregation (left) and ATP (right) release were displayed. (B) Washed platelets (2×10^8^ mL^-1^) were stimulated with indicated concentration of U46619. Percentage of platelet aggregation (left) and ATP (right) release were displayed. (C) Washed platelets (2×10^8^ mL^-1^) were stimulated with indicated concentration of ADP. Percentage of platelet aggregation was displayed. Statistical significance was evaluated with 2-tailed Mann-Whitney *U* test, the data are shown as median with interquartile range.


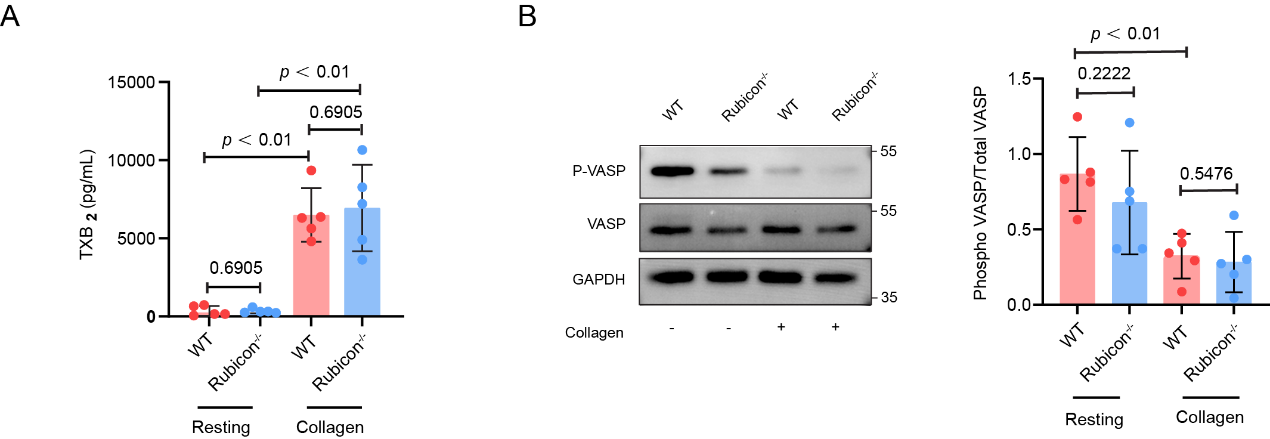


**Figure S3. Enhanced GPVI signaling in Rubicon^-/-^ platelets is independent of ADP/TXA₂ release.** (A) Platelet TXB_2_ released after collagen stimulation. (B) Western blot analysis of phosphorylated VSAP^S157^ in WT and Rubicon^-/-^ platelets after collagen stimulation. Quantification is shown as median with interquartile range (unpaired student *t* test).

**
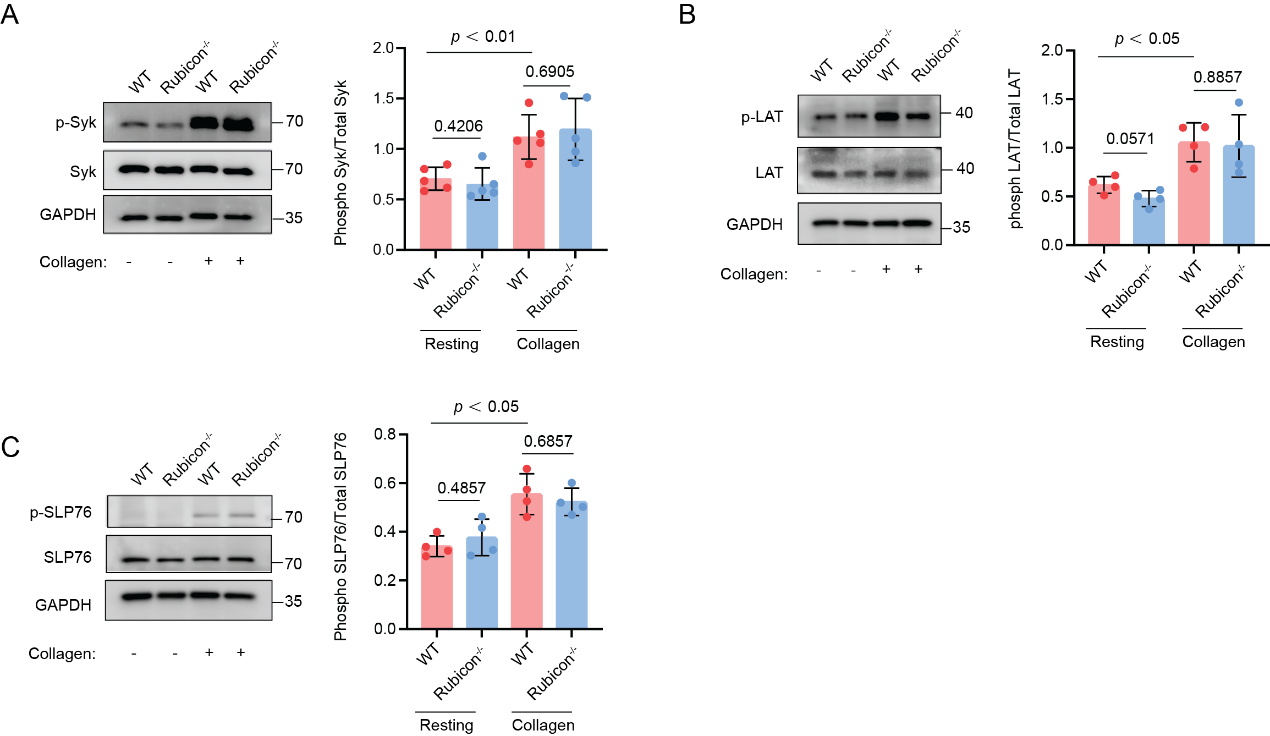
**

**Figure S4. Rubicon deficiency in platelet do not affect phosphorylation of Syk, LAT, and SLP-76.** Western blot analysis and quantification of phosphorylated Syk^Y352^, Lat^Y220^, SLP76^Y145^ in WT and Rubicon^-/-^ platelets stimulated with collagen. The results are shown as median with interquartile range (2-tailed Mann-Whitney *U* test).

**
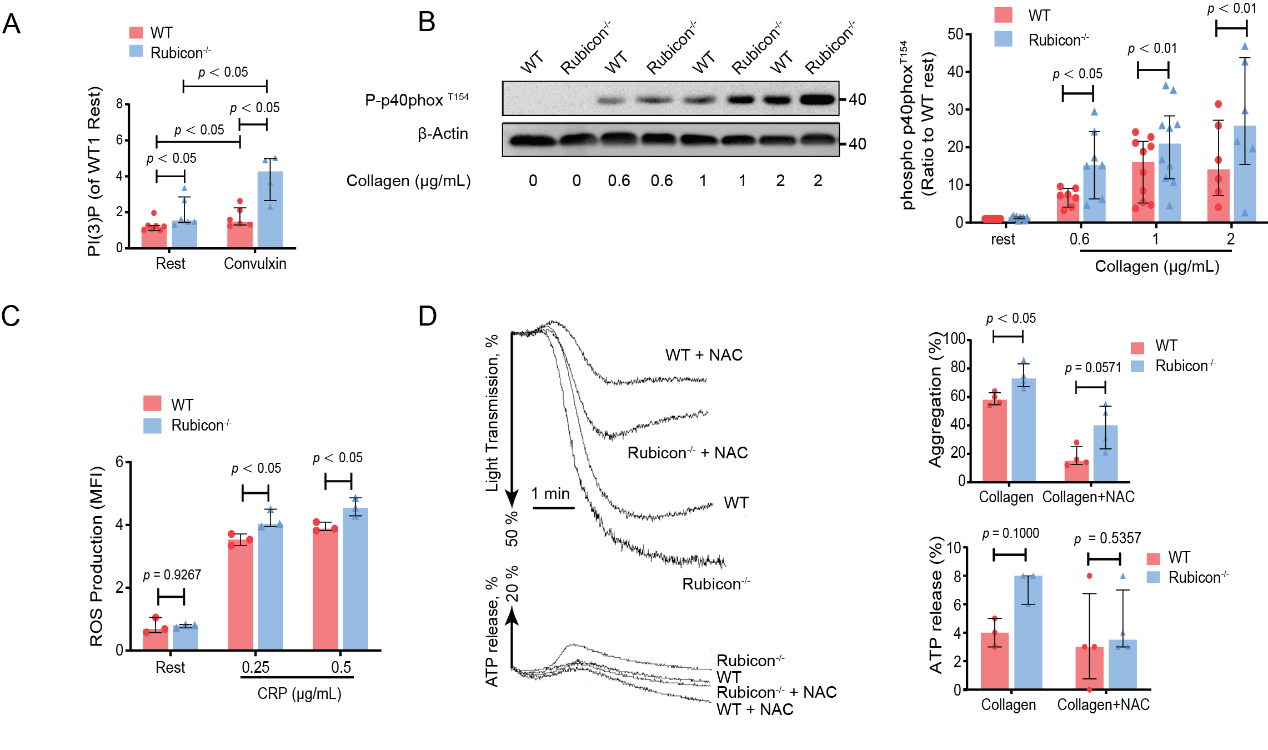
****Figure S5. ROS production is not accountable for Rubicon-contributed alteration of GPVI function.** (A) Phosphatidylinositol 3-phosphate (PI(3)P) levels during mice platelets activation. Samples were extracted from washed WT or Rubicon^-/-^ platelets (3×10^8^ mL^-1^), and PI(3)P production was measured using PI3P mass ELISA kit from at least 4 independent experiments (2-tailed Mann-Whitney *U* test). (B) Immunoblot analysis and quantification of p40-phox phosphorylation during WT and Rubicon^-/-^ platelets activation. Statistical significance was evaluated with student *t*-test. (C) ROS generation analyzed by flow cytometry. H_2_DCFDA (2’,7’-dichlorodihydrofluorescein diacetate)-loaded (50 µmol L^-1^) platelets were stimulated with 0.25 or 0.5μg mL^-1^ CRP for 5 min. Statistical significance was evaluated with unpaired student *t*-test. (D) Aggregation and ATP release of washed WT or Rubicon^-/-^ platelets stimulated with collagen (1 μg mL^-1^) in the presence or absence of NAC (1 mM) incubated for 5 minutes. Statistical significance was evaluated with 2-tailed Mann-Whitney *U* test. These data are shown as median with interquartile range.

**
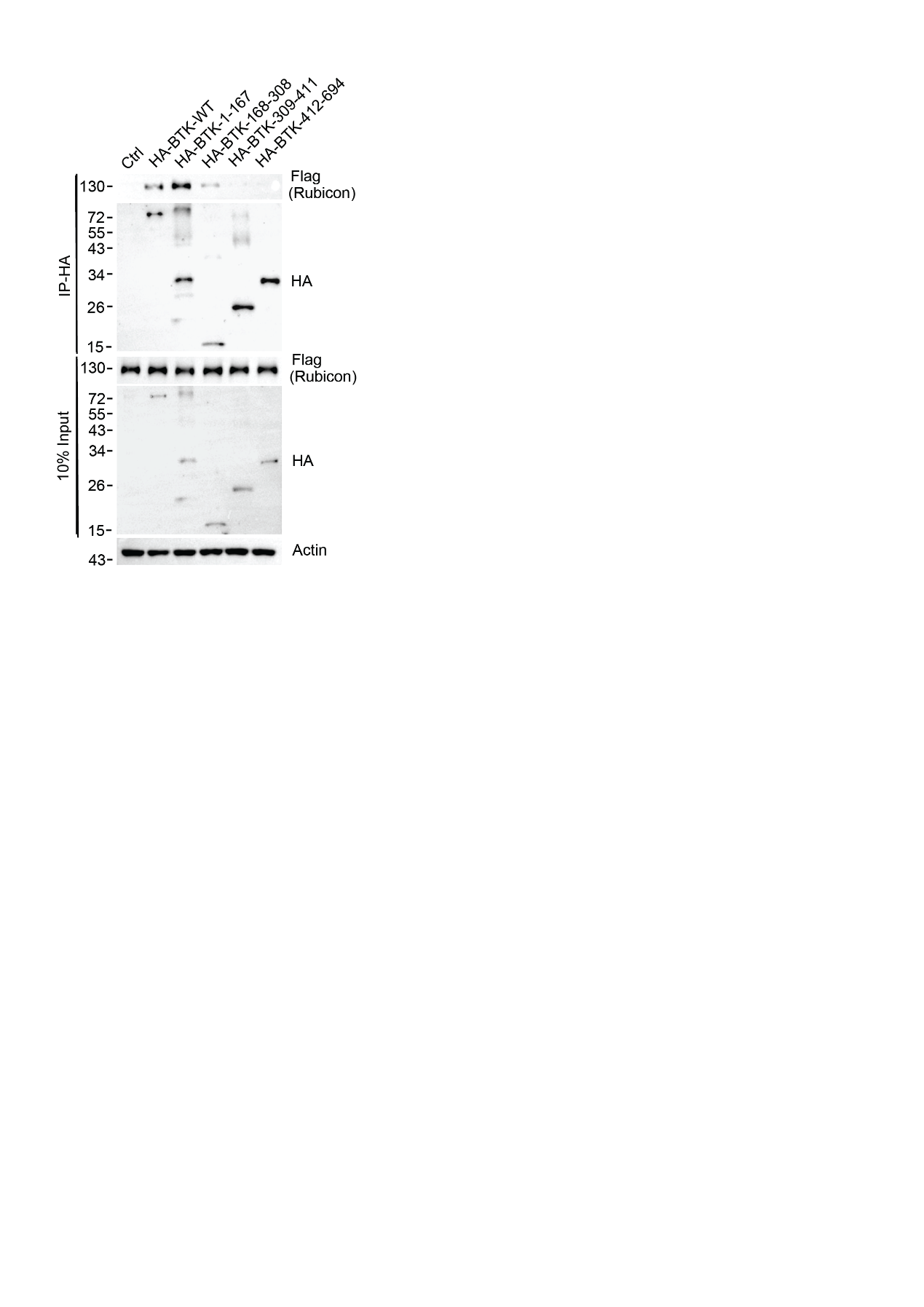
Figure S6. Rubicon interacts with Btk depending on the PH domain. of the Btk.** HA-tagged wild-type Btk and polypeptide fragments, including Btk aa 1-167, Btk aa 168-308, Btk aa 309-411, Btk aa 412-694 were co-expressed with FLAG-Rubicon in HEK293T cells. Cell extracts were immunoprecipitated with anti-HA antibody. The bound proteins or the 10% input extract was detected by immunoblotting using the antibodies indicated.


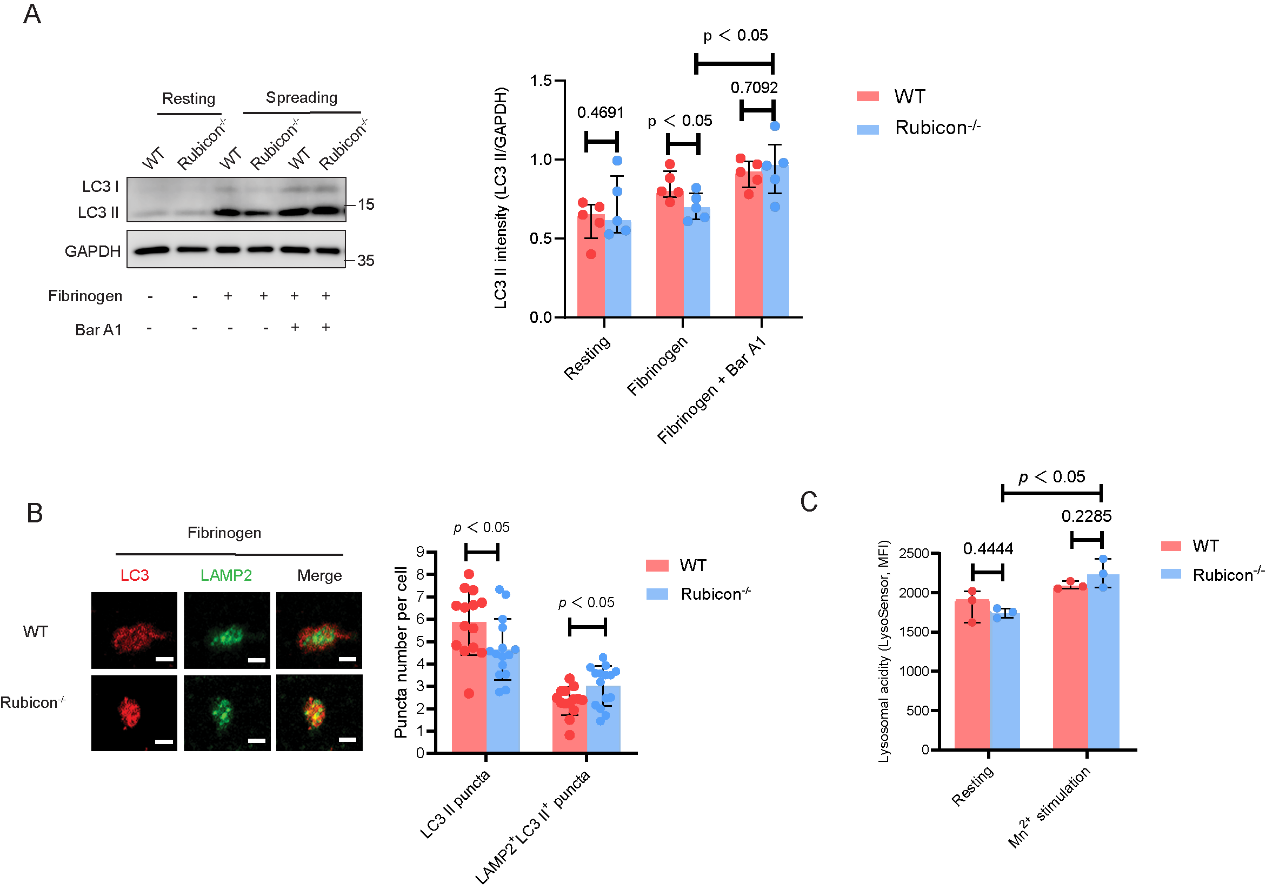
**Figure S7. Rubicon deficiency increases autophagosome-lysosome fusion but not lysosome acidity.** (A) LC3II protein levels in platelets after spreading on fibrinogen for 60 minutes in the presence or absence of Bar A1(500 nM). The results are shown as median with interquartile range (2-tailed Mann-Whitney *U* test). (B) Colocalization of LC3II puncta and LAMP2-labeled lysosome in platelets spread on fibrinogen (20 μg mL^-1^). Scale bar, 2 μm. (C) Flow cytometric analysis of platelet lysosome acidity after Mn^2+^ (0.5 mM) stimulation. The results are shown as median with interquartile range (2-tailed Mann-Whitney *U* test).


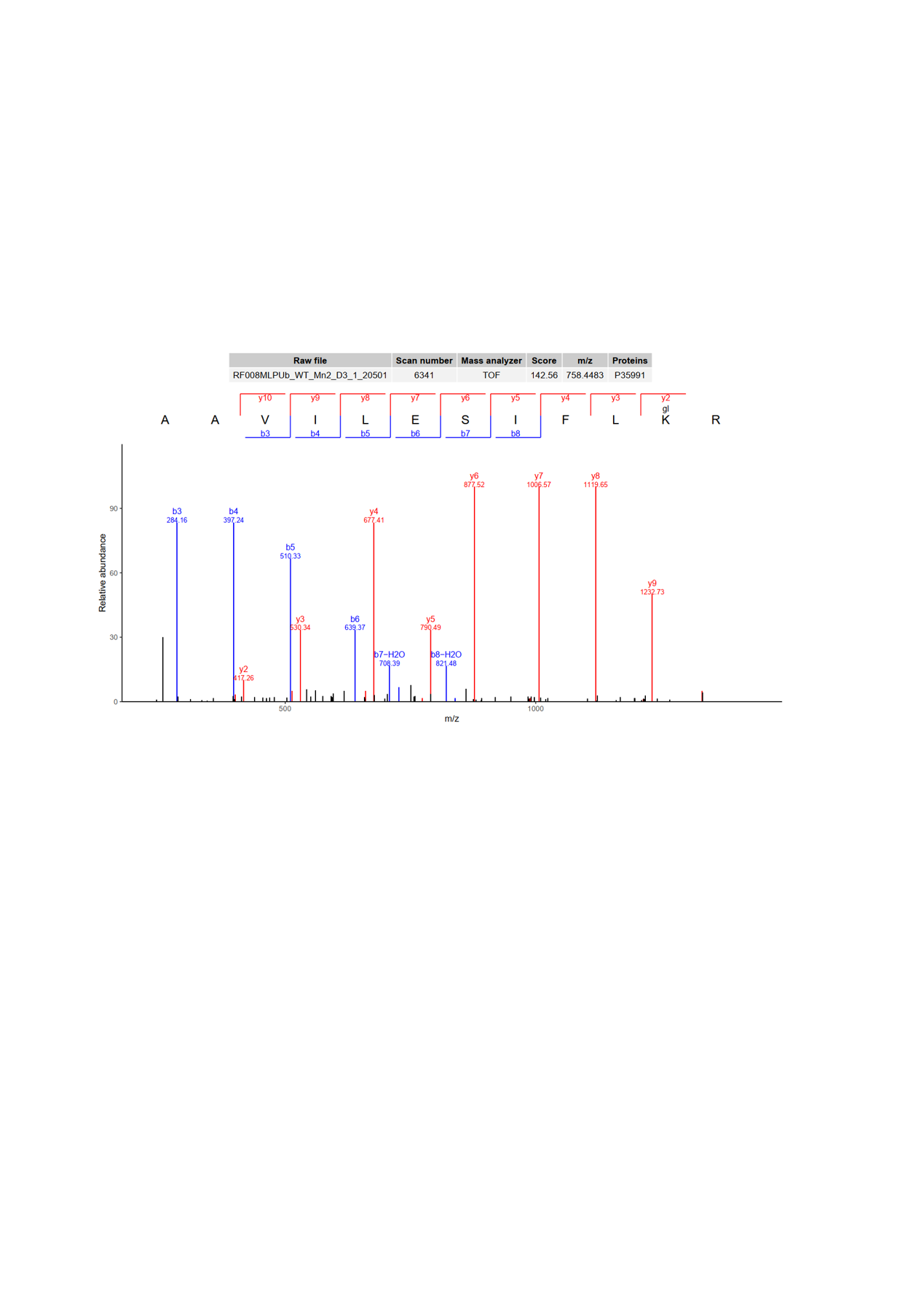
**Figure S8. Mass spectrum of Btk ubiquitination modification sites in WT Mn^2+^ stimulated platelet.**

**
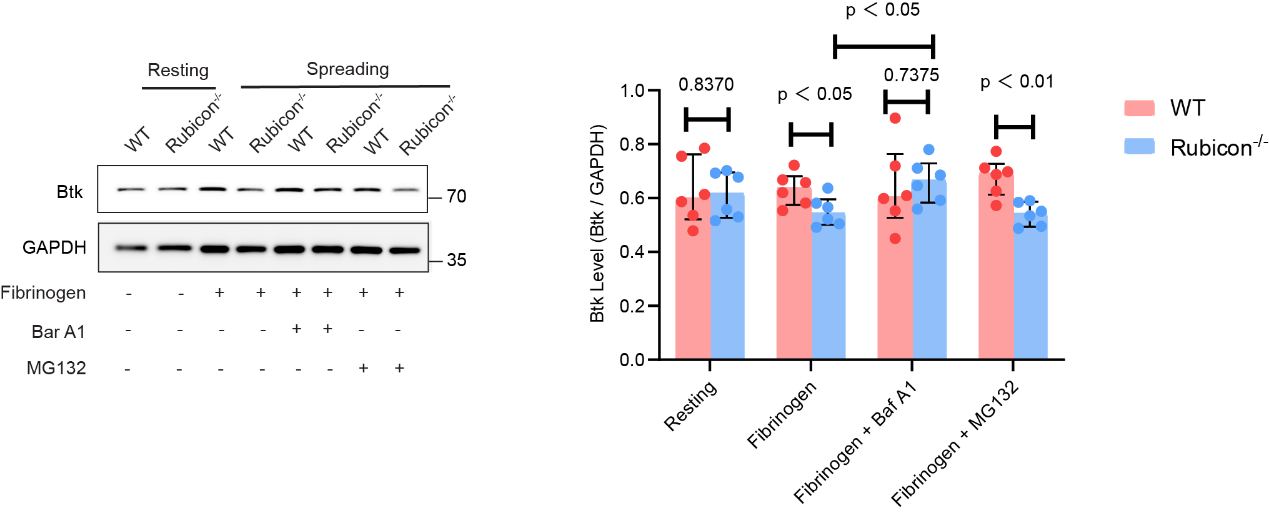
 Figure S9. Rubicon inhibits Btk degradation through the autophagy rather than the proteasome pathway.** BTK protein levels in platelets after spreading on fibrinogen for 60 minutes in the presence or absence of Bar A1(500 nM) or MG132 (30 μM). The results are shown as median with interquartile range (2-tailed Mann-Whitney *U* test).

**
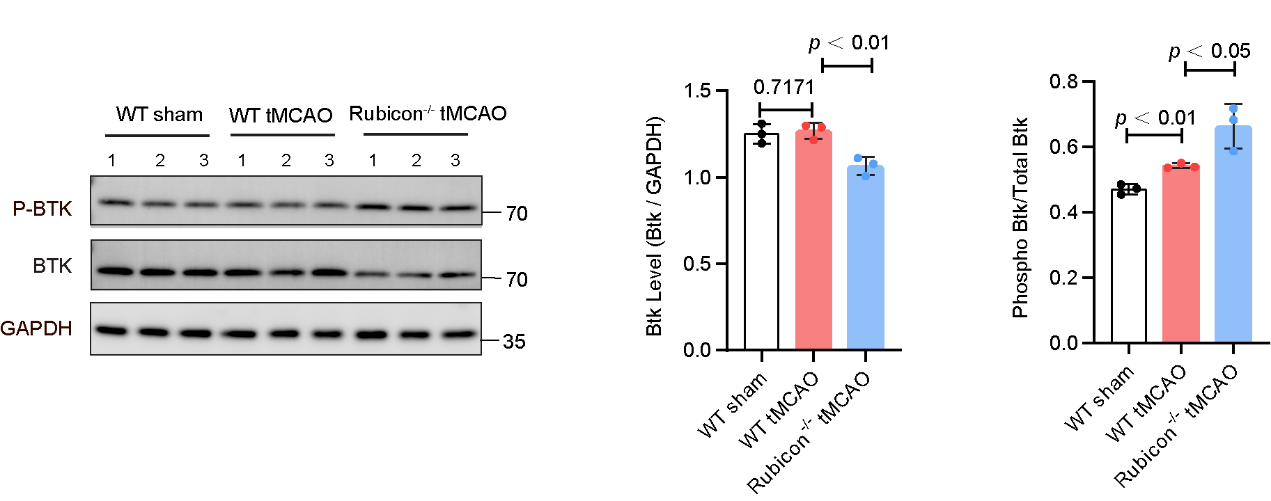
Figure S10. Rubicon inhibits Btk activation and degradation in platelets from tMCAO model.** Btk protein levels in platelets after spreading on fibrinogen for 60 minutes in the presence or absence of Bar A1(500 nM) or MG132 (30 μM) analyzed by immunoblot and quantified. The results are shown as median with interquartile range (2-tailed Mann-Whitney *U* test).

**Figure S11. Platelet Rubicon does not regulate infarct size during permanent middle cerebral artery occlusion.** *Rubcn^f/f^ PF4-Cre^+^* mice or littermate controls were subjected to permanent middle cerebral artery occlusion (24 hours, pMCAO). (A) Representative brain sections stained with 2,3,5-triphenyl-tetrazolium chloride. Healthy tissue stains red, while absence of staining indicates infarcted area. Scale bar, 1 cm. (B) Brain infarct volumes were quantified by planimetric analysis. The results are shown as median with interquartile range (2-tailed Mann-Whitney *U* test).**
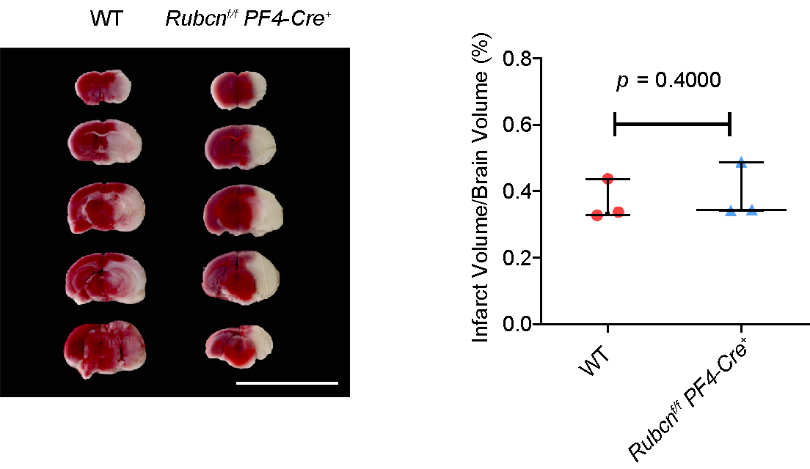
**


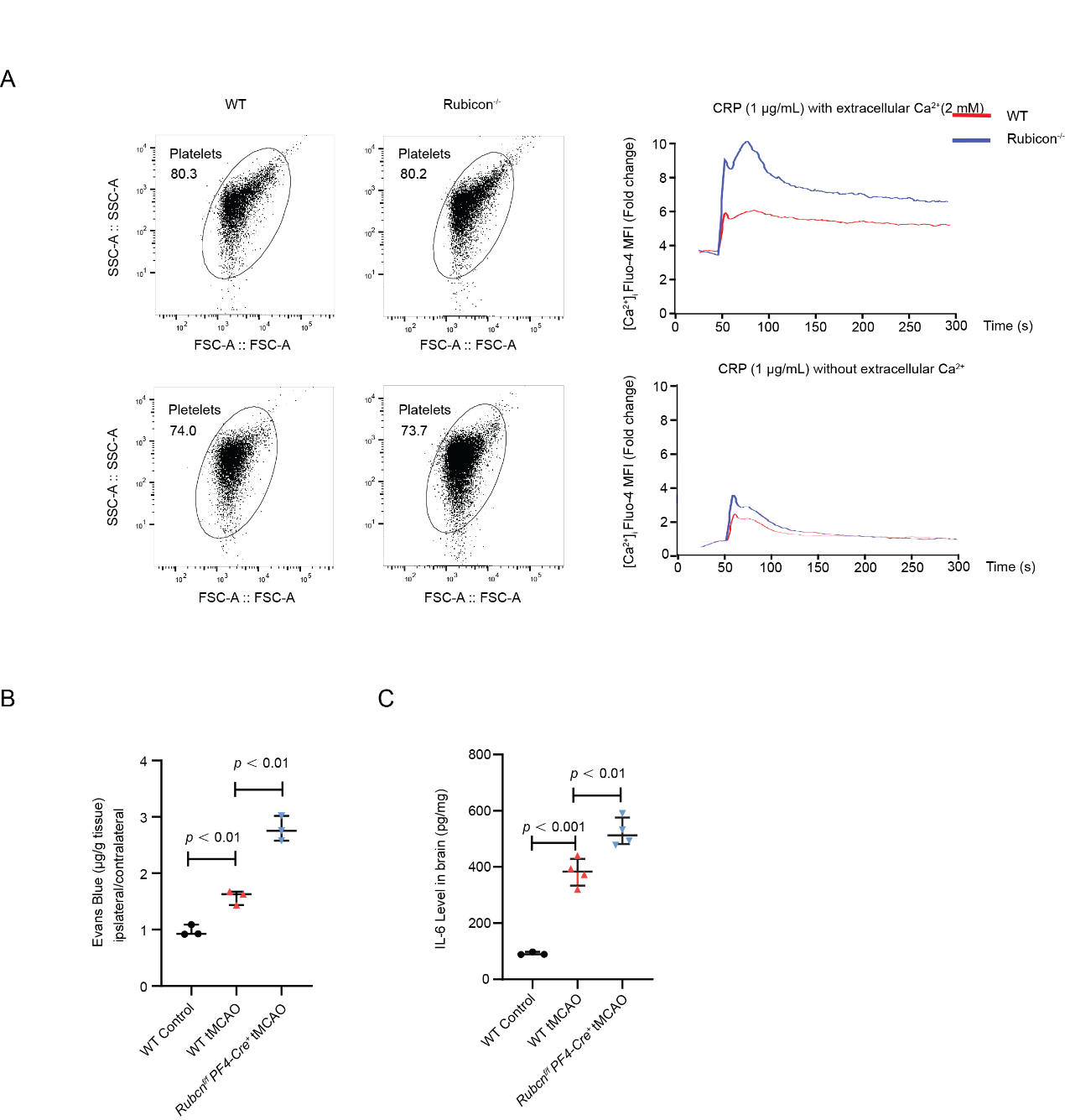


**Figure S12. Platelet Rubicon deficiency aggravates the secondary injury processes triggered by reperfusion in tMCAO model.** (A) Representative traces showing intracellular calcium flux in platelets in response to CRP (1μg/mL) stimulation. The kinetic traces represent three independent experiments. (B-C) *Rubcn^f/f^ PF4-Cre^+^* mice or littermate controls were subjected to tMCAO. Quantification of Evans Blue and IL-6 by ELISA of whole brain. The results are shown as median with interquartile range (unpaired students *t*-test).

**
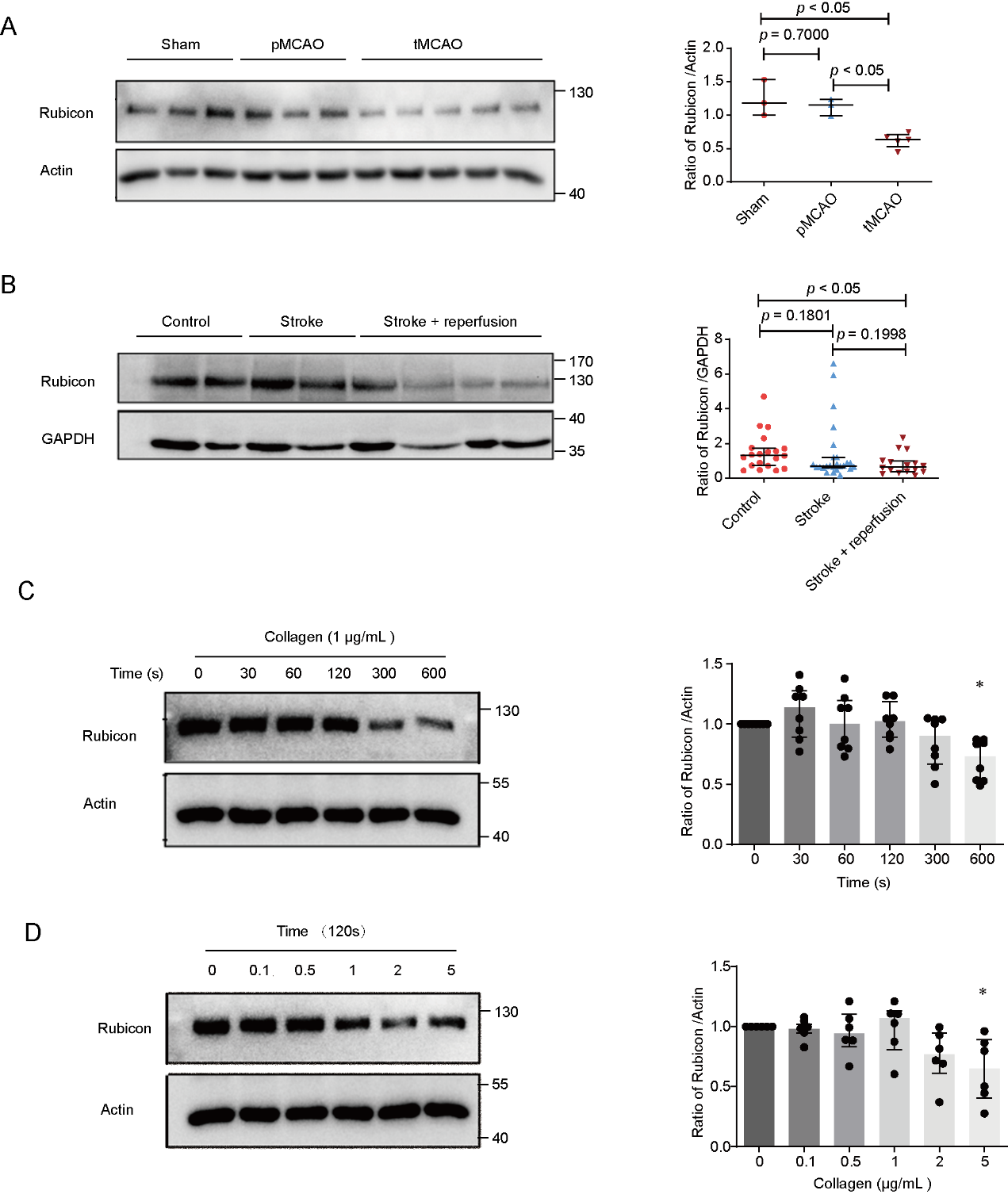
** **Figure S13. The expression of Rubicon in platelet decreased in tMCAO mice and in patients with acute ischemic stroke.** (A) Platelets from sham mice, pMCAO mice and tMCAO mice were lysed and analyzed level of Rubicon by Western blotting. Statistical significance was evaluated with 2-tailed Mann-Whitney *U* test. (B) Platelets from healthy control, stroke patients with or without thrombectomy, were lysed and analyzed level of Rubicon by Western blotting. Statistical significance was evaluated with 2-tailed Mann-Whitney *U* test. (C-D) Western blot analysis of Rubicon expression levels in platelets stimulated by collagen upon stimulation time or different concentration Statistical significance was evaluated with 1-way ANOVA test (**P* < .05). These results are shown as median with interquartile range.

**
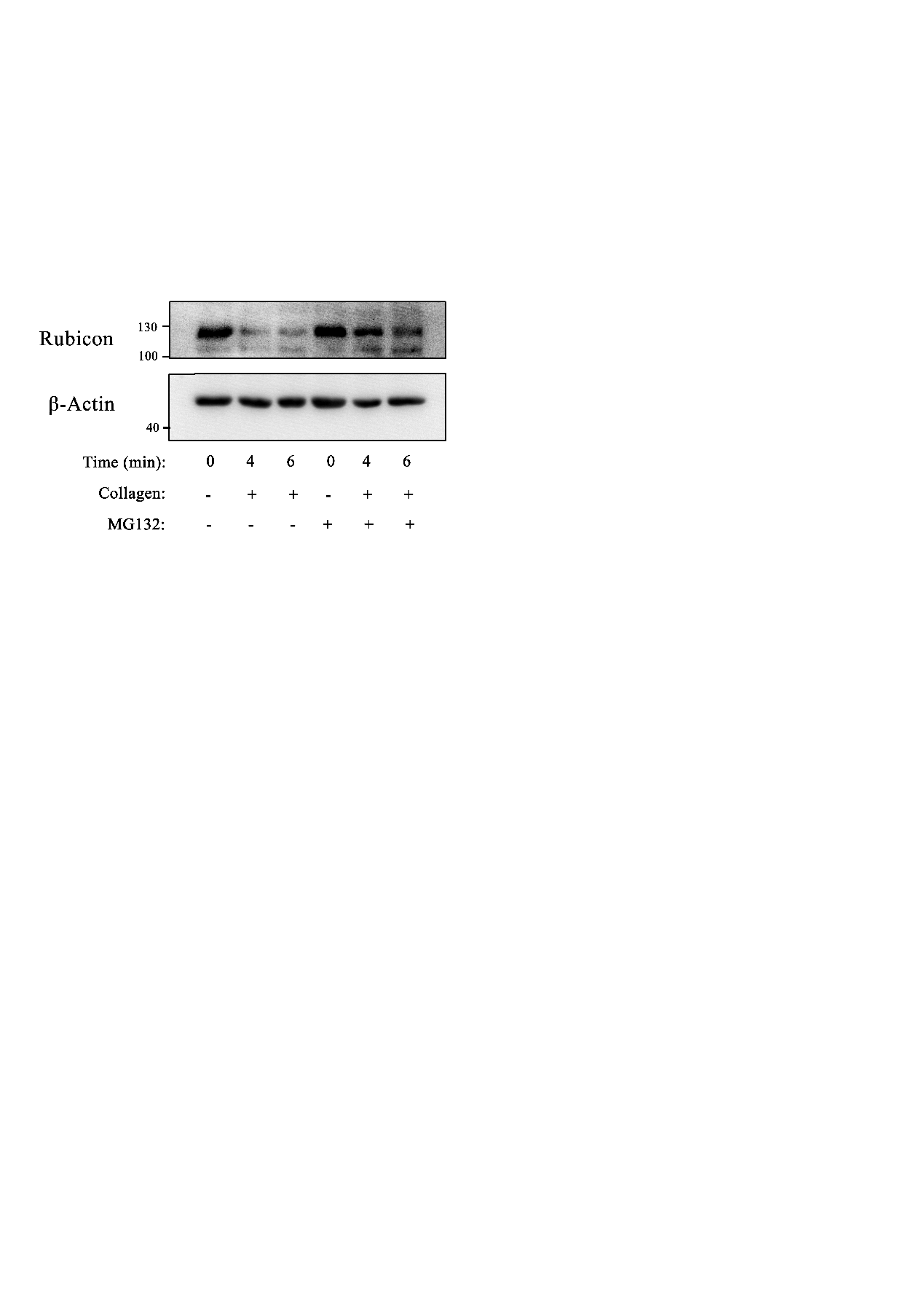
**

**Figure S14. Collagen introduced degradation of platelet Rubicon was inhibited by MG132.** Immunoblot analysis the level of Rubicon during collagen-induced WT platelets activation in the presence or absence of MG132 (20 μM).


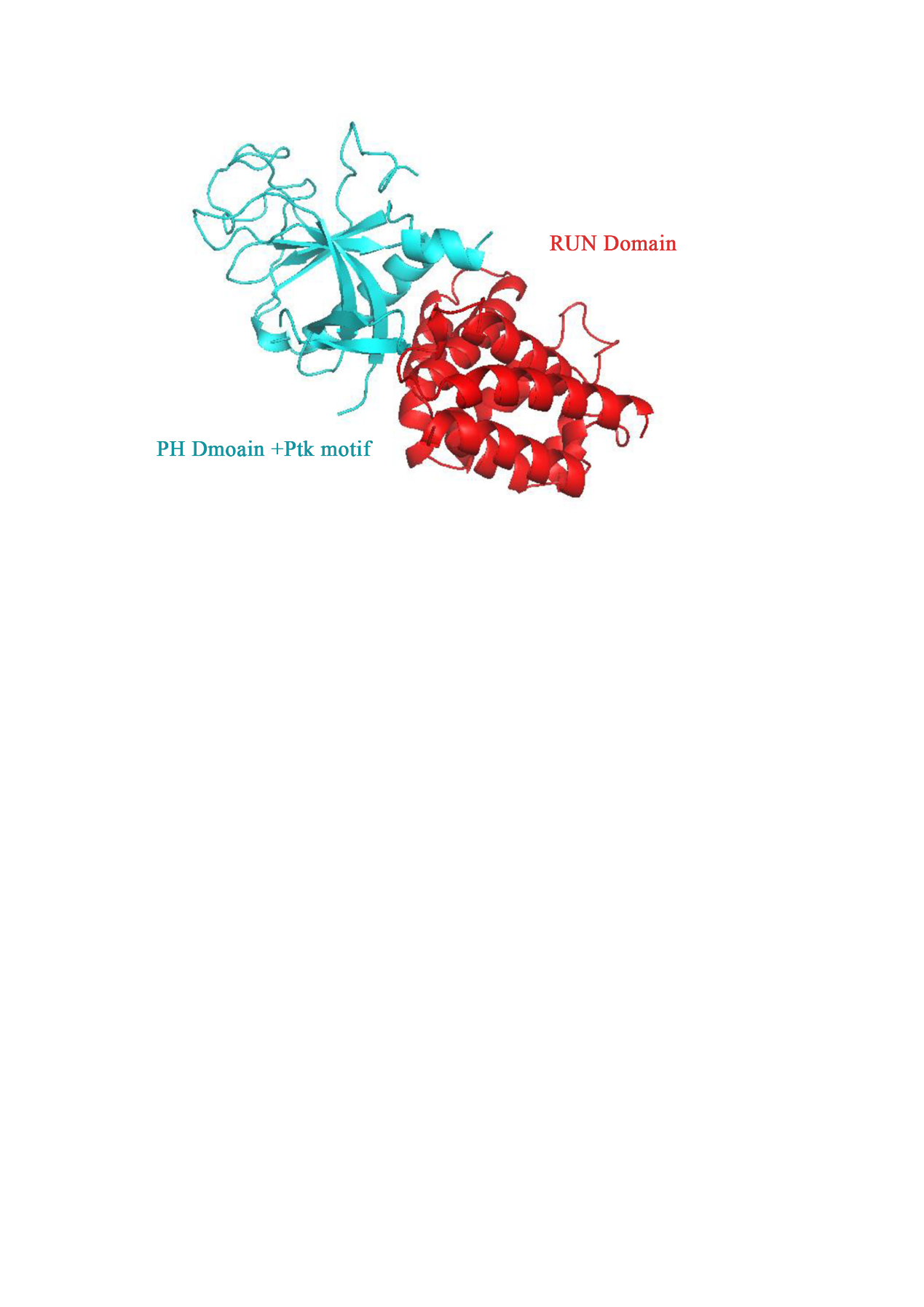
**Figure S15. The binding model of RUN domain of Rubicon to PH domain + Ptk motif of Btk.**

**Table S1. Proteins identified by Mass Spectrometry.**

**Table S2. Baseline characteristics of the patients and control (*n* = 61)****.**

**Table S3. Major entry Criteria.**

**Table S4. Hawkdock Scores for the top 10 docking models.**

**Table S5. Summary free binding energy for the top 10 models.**

**Video S1 *In vivo* imaging of thrombus formation following 10% FeCl_3_‐induced injury of mesenteric arterioles in *Rubcn^f/f^* control (WT) mice.**

**Video S2 *In vivo* imaging of thrombus formation following 10% FeCl_3_‐induced injury of mesenteric arterioles in *Rubcn^f/f^ PF4-Cre^+^* (Rubicon^-/-^) mice.**

**Video S3 Representative video of thrombus formation under flow condition *in vitro*.** Whole blood from WT mice was perfused through a collagen-coated surface in a microfluidic system at a shear rate of 300 s^-1^ for 5 min.

**Video S4 Representative video of thrombus formation under flow condition *in vitro*.** Whole blood from *Rubcn^f/f^ PF4-Cre^+^* mice was perfused through a collagen-coated surface in a microfluidic system at a shear rate of 300 s^-1^ for 5 min.

**Video S5 Representative video of thrombus formation under flow condition *in vitro*.** Whole blood from WT (upper panel) or *Rubcn^f/f^ PF4-Cre^+^* (lower panel) mice was perfused through a collagen-coated surface in a microfluidic system at a shear rate of 3000 s^-1^ for 5 min.
